# Evaluating a river's ecological health: A multidimensional approach

**DOI:** 10.1016/j.ese.2024.100423

**Published:** 2024-04-18

**Authors:** Qiuyun Zhao, Yangyang Zhang, Xiuwen Li, Xiaodong Hu, Rui Huang, Jixiong Xu, Zilong Yin, Xinjie Gu, Yuncheng Xu, Jinbao Yin, Qing Zhou, Aimin Li, Peng Shi

**Affiliations:** a State Key Laboratory of Pollution Control and Resource Reuse, School of Environment, Nanjing University, Nanjing, 210023, China; b Jiangsu Hydraulic Research Institute, Nanjing, 210023, China

**Keywords:** The lower reaches of the Yangtze River, High-risk emerging contaminants, Plankton, Ecological risk, Biological integrity

## Abstract

Evaluating the health of river surface water is essential, as rivers support significant biological resources and serve as vital drinking water sources. While the Water Quality Index (WQI) is commonly employed to evaluate surface water quality, it fails to consider biodiversity and does not fully capture the ecological health of rivers. Here we show a comprehensive assessment of the ecological health of surface water in the lower Yangtze River (LYR), integrating chemical and biological metrics. According to traditional WQI metrics, the LYR's surface water generally meets China's Class II standards. However, it also contains 43 high-risk emerging contaminants; nitrobenzenes are found at the highest concentrations, representing 25–90% of total detections, while polycyclic aromatic hydrocarbons present the most substantial environmental risks, accounting for 81–93% of the total risk quotient. Notably, the plankton-based index of biological integrity (P-IBI) rates the ecological health of the majority of LYR water samples (59.7%) as ‘fair’, with significantly better health observed in autumn compared to other seasons (*p* < 0.01). Our findings suggest that including emerging contaminants and P-IBI as additional metrics can enhance the traditional WQI analysis in evaluating surface water's ecological health. These results highlight the need for a multidimensional assessment approach and call for improvements to LYR's ecological health, focusing on emerging contaminants and biodiversity rather than solely on reducing conventional indicators.

## Highlights

• We suggest a multidimensional approach to evaluate a river's ecological health.
• PAHs are most crucial among 43 high-risk emerging contaminants.
• 59.7% of the surface water samples are in a “fair” state.
• Emerging contaminants and biodiversity are vital for ecological health assessment.

## Graphical abstract

**Figure:**
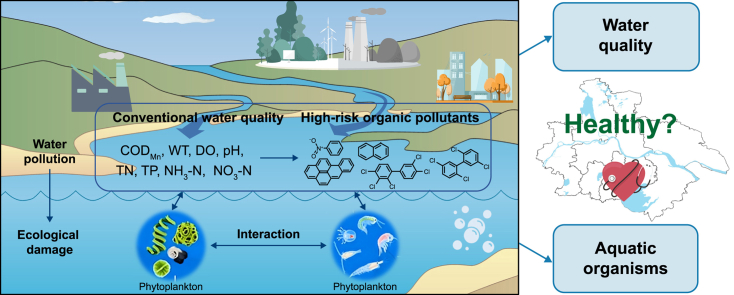

## 1 Introduction

Evaluating river surface water health status is crucial, because rivers usually harbor significant biological resources and are critical drinking water sources [1]. Currently, the widely employed method for comprehensively evaluating the water environment is the water quality index (WQI) [[2], [3], [4]]. Wu et al. [5], for instance, evaluated the water quality of Taihu Lake using the WQI method and indicated an overall water quality rating at a “medium” level (The scale is 51–70 on a total scale of 100). Moreover, the risk quotient (RQ) is a quantitative evaluation method that combines exposure levels of detected chemicals with hazard assessments [6]. It has found extensive application in the environmental risk assessment of organic compounds [[7], [8], [9]].

Emerging contaminants, such as polychlorinated biphenyls (PCBs), polycyclic aromatic hydrocarbons (PAHs), and nitrobenzenes (NBs), are known for their high toxicity, persistence and ease of migration [10,11]. Before they were banned in 1974, China was one of the world's major producers and consumers of PCBs [12]. These past-used PCBs are still present in the environment today, causing reproductive and immune deficiencies, endocrine disorders, neurodevelopmental dysfunction, and cancer in organisms [13]. Besides natural sources, PAHs mainly come from anthropogenic combustion processes, such as fossil fuel combustion and garbage incineration [14]. Prolonged exposure to PAHs can delay the growth and development of aquatic organisms, resulting in reproductive and immune defects, endocrine disorders, and neurodevelopmental disorders [15,16]. NBs are important raw chemical materials or intermediates widely used in fuels, explosives, pesticides, and polymer chemical industries [17] and can result in nervous system abnormalities, anemia, liver diseases, etc. [17,18]. These high-risk emerging contaminants are widely distributed in surface water presently, including main streams [19,20], tributaries [21], lakes [22], and even drinking water sources [23]. Hence, it is imperative to characterize their presence and associated risk.

However, the watershed water ecosystem is notably complex, resulting in the inability to exhaust all environmental factors. Biological indicators offer insights into environmental changes by observing the responses of aquatic organisms to stressors [24]. Thus, the index of biological integrity (IBI) has become a vital method for assessing the overall health of water ecosystems, as it synthesizes various biological indicators [[25], [26], [27]]. Plankton, as the foundation of the aquatic food web, exerts a profound influence on the growth and reproduction of other aquatic organisms, making it essential for the proper functioning of ecosystems and the provision of ecological services [28,29]. They are typically small, have a relatively short lifespan, and are sensitive to environmental changes [24]. Therefore, the Plankton-based Index of Biological Integrity (P-IBI), and the related parameters such as species composition, biomass, abundance, and plankton diversity are frequently employed to characterize the changes in water quality [30].

The Yangtze River, China's largest and the world's third-largest river, spans 6397 km with an annual runoff of about 960 billion m^3^. The lower reaches of the Yangtze River (LYR) refer to the stretch from the Nanjing and Anhui junction to the Yangtze River estuary, one of China's most important economic belts (Yangtze River Delta Economic Belt). However, economic development has led to a continuous influx of 17.8 billion tons of wastewater annually into the LYR. This has resulted in serious water pollution, posing a significant threat to aquatic life [7]. Currently, the water quality evaluation of the LYR is mostly based on WQI as the main research method, and evaluating aquatic biological integrity is mostly focused on lakes or reservoirs, but less research has been reported in the mainstream. Additionally, few reports have discussed the relationship between the evaluation results of the chemical indicators and aquatic biological indicators. Therefore, we suppose that the chemical indicators were insufficient to evaluate the real quality of the surface water. To verify our hypotheses, our investigation encompassed the spatiotemporal distribution analysis of conventional water quality indices, three types of high-risk emerging contaminants, and the plankton communities. Then, we comprehensively evaluated the water's ecological health by integrating the chemical and biological indicators. Eventually, we compared the outcomes of the three evaluation methods to gain insights into their differences. The findings of this study hold the potential to provide a robust theoretical foundation for the comprehensive management and ecological protection efforts of the LYR.

## 2 Materials and methods

### 2.1 Standards and reagents

The standard mixture solutions for 16 PCBs, 13 PAHs, and 14 NBs were purchased from o2si (Charleston, South Carolina, U.S.), with each standard chemical boasting a purity level exceeding 98%. Methanol, dichloromethane, ethyl acetate, and *n*-hexane, all of chromatographic purity, were sourced from Sigma Aldrich Inc (Saint Louis, MO, U.S.). Lugol's solution was supplied by Shanghai Yuanye Bio-Technology Co., Ltd (Shanghai, China). The formaldehyde solution was purchased from Aladdin (Shanghai, China). Detailed information about the reagents is listed in Table S1.

### 2.2 Sample collection

**Fig. 1:**
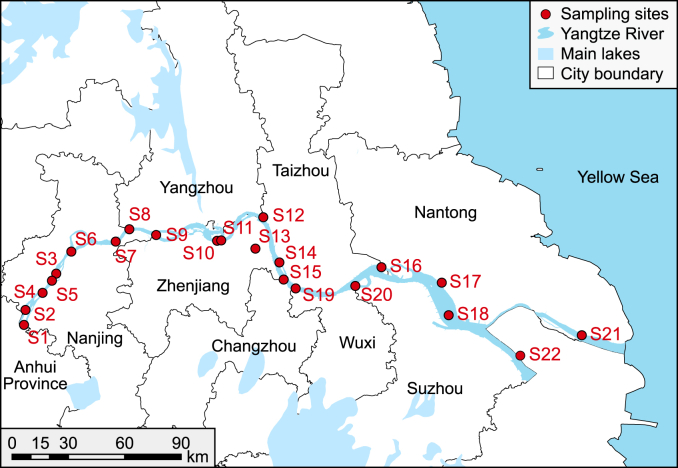
Sampling locations in the LYR. According to the geographical distribution characteristics, the study area was divided into the upper reaches (S1–S7), the middle reaches (S8–S15 and S19), and the lower reaches (S16–S18 and S20–S22).

Four sampling campaigns were conducted in January, April, July, and November 2021. Twenty-two samples were collected in each season, covering eight cities along the LYR, including national reserves and drinking water sources. Detailed sampling strategies are shown in Text S1. However, the sample at site 18 in November was not collected due to the COVID-19 pandemic, resulting in a gap in the data. The specific sampling site distribution is shown in Fig. 1 and Table S2.

Five liters of water samples at each sampling site were collected by a stainless steel bucket about 20 m from the shore and 1 m below the surface. The bucket was then placed in a clean brown glass bottle to keep in the dark.

0.5-liter samples of phytoplankton and Rotifera were collected from each cross-section's surface and middle layers. These samples were combined and placed into a 1-L sampling bottle after collection. Then we fixed them using 1% (V/V) Lugot's solution to preserve them. For Cladocera and Copepoda, we collected 20 L of mixed samples. These samples were then filtered through a No. 25 planktonic net and concentrated to a constant volume of 50 mL. Finally, the concentrated samples were stored in a 5% (V/V) formaldehyde solution for further testing. All samples were immediately transported back to the laboratory and pretreated within 24 h.

Regarding plankton samples, sampling is only conducted within a small range and following standard requirements, ensuring it does not affect the biodiversity of the Yangtze River.

### 2.3 Analytical methods

Water temperature (WT), pH, and dissolved oxygen (DO) were measured by a portable multiparameter meter (HACH, SL 1000, U.S.) in the field. Permanganate index (COD_Mn_), total phosphorus (TP), total nitrogen (TN), ammonia nitrogen (NH_3_-N), and nitrate nitrogen (NO_3_-N) were determined according to the latest standard method in China. Specific laboratory methods are displayed in Text S2.

The pre-treatment steps for high-risk emerging contaminant samples, including filtration, acid regulation, extraction, and concentration, are detailed in Text S3. Then, the detection was performed on a gas chromatography-mass spectrometry (GC-MS, Thermo Scientific, Trace GC Ultra-ISQ, U.S.) with a DB-5/MS column (Agilent, 30 m × 0.25 mm × 0.25 mm, U.S.). The GC and MS conditions of PCBs, PAHs, and NBs are shown in Table S3, and the details of their retention times and qualitative and quantitative ions are listed in Table S4. The quality assurance and quality control of high-risk emerging contaminants are shown in Text S4, and the details for the method detection limit and recovery rates are displayed in Tables S5–S7.

The zooplankton and phytoplankton were identified and counted by the Nanjing Haoan Environmental Monitoring Co., Ltd. The detailed calculation formula of the dominance index (Y) is displayed in Text S5.

### 2.4 Water quality assessment

The calculation formula for WQI [9] is shown in text S6. The interpretation of WQI values is as follows: WQI ≤ 0.25 indicates clean water, 0.25 < WQI ≤ 0.50 suggests relatively clean water, 0.50 < WQI ≤ 0.75 indicates light pollution, 0.75 < WQI ≤ 0.99 signifies medium pollution, and WQI = 1.0 represents severe pollution.

The RQ is the ratio of predicted environmental concentration (PEC) or measured environmental concentration (MEC) to the predicted non-effective concentration (PNEC) of a compound [31]. The calculation process is detailed in Text S7, and the PNEC values for the 43 compounds used in this study are presented in Table S8. Typically, RQ values are interpreted as follows: RQ < 0.01 indicates no risk, 0.01 ≤ RQ < 0.1 suggests low risk, 0.1 ≤ RQ < 1.0 indicates medium risk, and RQ ≥ 1.0 represents high risk [32].

### 2.5 P-IBI calculation

The establishment of the plankton-based IBI followed the IBI evaluation system proposed by Zhang et al. [33]. Initially, reference points and impaired points needed to be determined. In this study, sample points with favorable water environment quality (WQI < 0.5) and rich biodiversity (Shannon-wiener index ≥ 2) were selected as reference points [34]. According to this criterion, 15 reference points and 72 impaired points were chosen to construct the P-IBI evaluation system. More details are available in Table S9.

Subsequently, 41 candidate biological indicators, which have been used worldwide for plankton to effectively evaluate the ecological environment of surface water [33,35,36], were screened. This screening involved a distribution range test, discrimination ability test, and correlation analysis [37]. The specific steps are presented in Text S8. The boxplot illustrating each indicator's reference points and impaired points is shown in Fig. S1, and the results of the Pearson correlation analysis are provided in Table S10. Ultimately, 13 parameters were selected as the core indicators for the LYR's P-IBI system.

The calculation process for P-IBI is described in Text S9, and the calculation formula for the 13 core indicators is shown in Table S11. The classification of evaluation levels adopted the method of four equal parts. The interpretation of each level is as follows: P-IBI ≥ 1 indicates excellent water quality, 0.75 ≤ P-IBI < 1 suggests good quality, 0.5 ≤ P-IBI < 0.75 signifies fair quality, 0.25 ≤ P-IBI < 0.5 implies poor quality, and P-IBI < 0.25 represents extremely poor water quality [33].

### 2.6 Data analysis

SPSS Statistic 26.0 software was used for one-way analysis of variance (ANOVA) and Pearson correlation analysis. Shannon-Wiener diversity index (H′), Pielou evenness index (J), and Margalef richness index (d) were calculated using Past 3 software. Applying Canoco 5 software for canonical correspondence analysis (CCA) and redundancy analysis (RDA) analysis. WQI, RQ, and P-IBI were calculated using Excel 2019 according to the formula. Origin 2021 and ArcGIS 10.6 software were used for drawing.

## 3 Results and discussion

### 3.1 Spatiotemporal distribution of high-risk emerging contaminants

**Fig. 2:**
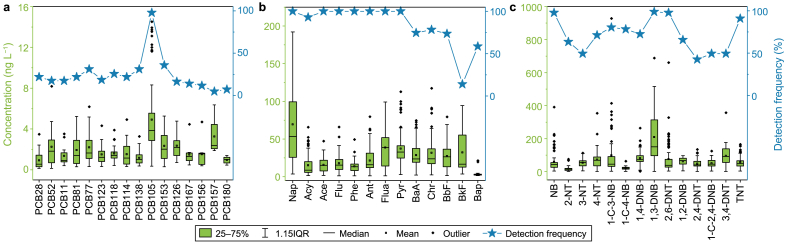
The detected concentrations and frequencies of PCBs (**a**), PAHs (**b**), and NBs (**c**) in the LYR. The boxplot represents the concentration ranges, while the line charts depict the detection frequencies.

All 43 compounds were detected in the LYR, with a total concentration ranging from 173.19 to 3853.46 ng L^−1^. The concentrations and detection frequencies of these compounds are depicted in Fig. 2. Notably, NBs constituted a significant proportion, accounting for concentrations between 25.0% and 89.7%, with an average concentration significantly higher than that of other compounds (*p* < 0.01). PAHs were the second most prevalent group, ranging from 10.2% to 71.6%. In contrast, PCBs constituted only a minor percentage of concentrations, ranging from 0.1% to 3.4% (Fig. S2). In comparison with previous studies, the concentrations of PCBs, PAHs, and NBs measured in this study were found to be on par with those in rivers in eastern China, such as the Huaihe River [21], the Qiantang River [38], and the Songhua River [39]. They were marginally higher than concentrations reported in developed countries like Russia [40], Germany [41], and Italy [42] but significantly exceeded levels found in less developed regions like Egypt [43] and Cuba [44]. For a comprehensive view of the global distribution of PCBs, PAHs, and NBs in surface water, refer to Table S12 in the supplementary materials.

These organic pollutants exhibited diverse distribution patterns across different seasons. Summer showed the highest diversity in the types of compounds detected, surpassing other seasons. During this period, the total concentration ranged from 1174.23 to 3853.46 ng L^−1^ (Fig. S3). Winter followed, with concentrations varying from 173.19 to 2512.36 ng L^−1^, and autumn displayed a range from 703.16 to 1457.85 ng L^−1^. The significance of differences in the temporal distribution of these organic pollutants is displayed in Fig. S4a. This finding aligns with the results of Zhao et al. [20]. The increase in rainfall during the wet season facilitates the entry of pollutants into water bodies through surface runoff. This has a dual effect: it directly increases the influx of organic matter into the water while also inducing significant fluctuations in water volume, which can impair the efficiency of sewage treatment plants. Consequently, this reduces pollutant removal and reduces effluent quality [45]. Furthermore, in summer, when external environmental conditions change or are influenced by organisms, organic pollutants that have adsorbed into sediment can be released back into the water, causing secondary pollution [46].

In terms of spatial distribution, although the spatial difference is not significant (Fig. S4b), the overall trend of the total concentration of the organic pollutants was: downstream (the average value was 1369.42 ng L^−1^) > midstream (1252.69 ng L^−1^) > upstream (1142.19 ng L^−1^). This spatial variation can be attributed to pollutants generated by human activities along the river, which flow downstream with the river's current, thereby contributing to increased concentrations in downstream areas [47]. Notably, the highest annual average concentration was recorded in the downstream section of Suzhou (S22), reaching 1684.62 ng L^−1^. Our field investigation revealed that this section is near the Suzhou chemical industry park and several shipping terminals. Previous studies have shown that scraping metal from abandoned ships, especially those coated with paint, can release higher levels of PCBs into the environment. Additionally, shipping-concentrated areas often exhibit elevated concentrations of PAHs [48]. Along the LYR, there are numerous dye factories in regions such as Zhenjiang, Taizhou, Changzhou, and Suzhou, which are likely significant sources of NBs in the LYR [49].

### 3.2 Composition and source analysis of high-risk emerging contaminants

**Fig. 3:**
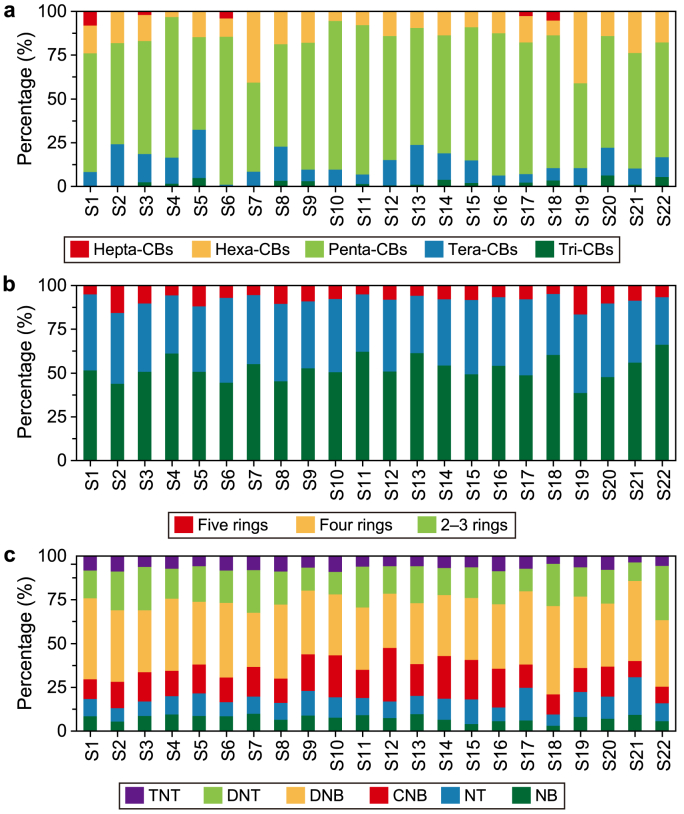
Composition of PCBs (**a**), PAHs (**b**), and NBs (**c**). According to the different structural characteristics, PCBs were divided into Tri-CBs, Tera-CBs, Penta-CBs, hexachlorobiphenyls (Hexa-CBs), and heptachlorobiphenyls (Hepta-CBs); PAHs were divided into 2–3, four, and five rings; and NBs were divided into nitrobenzene (NB), nitrotoluene (NT), chloro-dinitrobenzene (CNB), dinitrobenzene (DNB), dinitrotoluene (DNT), and trinitrotoluene (TNT).

The primary contributors to PCBs were pentachlorobiphenyls (Penta-CBs), accounting for 49–85% of the total, with concentrations ranging from 2.17 to 9.45 ng L^−1^ (Fig. 3a). Overall, low-chlorinated biphenyls, including trichlorobiphenyls (Tri-CBs), tetrachlorobiphenyls (Tera-CBs), and Penta-CBs constituted 59–97% of the total PCBs in this study. This phenomenon seems linked to the higher water solubility of low-chlorinated biphenyls compared to highly-chlorinated biphenyls [23]. Additionally, PCBs produced and used in China were predominantly low-chlorinated biphenyls from 1965 to 1974, aligning with the composition of PCBs detected in this study [50]. This suggests that PCBs in the LYR primarily originate from historical production and use. As shown in Fig. 3b, PAHs of 2–3 rings were the most dominant, constituting 39–66% of the total PAHs, followed by four rings (27–48%) and five rings (5–17%). This distribution may be attributed to the low molecular weight (LMW), which exhibits lower log *K*_OW_ values and greater water solubility [9]. DNB accounted for the largest proportion of NBs, ranging from 31% to 50%, followed by dinitrotoluene (DNT, 11–31%) and chloro-dinitrobenzene (CNB, 9–31%) (Fig. 3c). Environmental NBs primarily stem from wastewater and flue gas emissions from chemical plants and dye factories. Particularly, aniline dye factory effluents are known to contain substantial quantities of NBs.

Isomeric ratios were applied to analyze the source of PAHs in the environment [51]. In spring, the ratios of Fla/(Fla + Pyr) were mostly less than 0.4, indicating that petroleum sources may be the primary contributors to PAHs (Fig. S5a). However, during summer, autumn, and winter, the ratios of Fla/(Fla + Pyr) exceeded 0.4, manifesting that the PAHs primarily originated from combustion. Furthermore, the type of combustion was differentiated, as depicted in Fig. S5b. The results revealed that biomass and coal combustion were the primary sources of PAHs in autumn and winter. Nevertheless, the PAHs in summer were derived from mixed sources of petroleum, biomass, and coal combustion. This variation in summer may be attributed to increased shipping and vehicle activities during the wet season, resulting in PAHs from petroleum combustion entering the water body [52].

### 3.3 Characteristics of plankton community structure

A total of 81 phytoplankton species, spanning six phyla, eight classes, 18 orders, 29 families, and 50 genera, were identified in this study. The dominant phyla were Bacillariophyta, comprising 33 species (40.7%), and Chlorophyta, comprising 29 species (35.8%). In general, there was a slightly higher diversity of phytoplankton in autumn, with 55 species, compared to spring (50 species), summer (50 species), and winter (48 species), but the difference was not significant.

The annual average phytoplankton abundance in the LYR was 1.38 × 10^6^ individuals per liter (ind. L^−1^). Bacillariophyta exhibited the highest abundance, accounting for 49.4%, followed by Cyanophyta (26.7%). These findings are consistent with a previous study by Wu et al. [53], which reported that the phytoplankton community in the middle and lower reaches of the Yangtze River was dominated by Bacillariophyta. Bacillariophyta tends to be the dominant group in large rivers, contributing over 60% of the density and biomass [54]. This is due to Bacillariophyta's ability to thrive in various challenging environmental conditions, including low temperatures, high salinity, and high alkalinity [55]. During this research, the WT ranged from 13.1 to 29.2 °C in spring, summer, and autumn, and pH ranged from 7.21 to 8.54. These conditions are particularly favorable for the growth of Bacillariophyta.

**Fig. 4:**
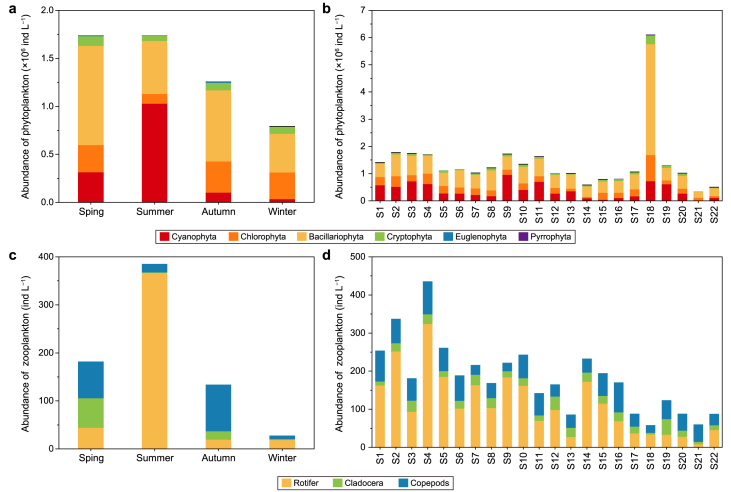
Temporal and spatial variation of plankton abundance. **a**, Temporal distribution of phytoplankton abundance; **b**, Spatial distribution of phytoplankton abundance; **c**, Temporal distribution of zooplankton abundance; **d**, Temporal distribution of zooplankton abundance.

The spatiotemporal distribution of phytoplankton abundance is plotted in Fig. 4a and b. Regarding the temporal aspect, there was a noticeable seasonal variation (*p* < 0.05), with an overall pattern of spring (1.74 × 10^6^ ind. L^−1^) ≈ summer (1.74 × 10^6^ ind. L^−1^) > autumn (1.26 × 10^6^ ind. L^−1^) > winter (0.79 × 10^6^ ind. L^−1^). This pattern corresponds with temperature changes. Temperature plays a significant role in regulating phytoplankton growth by influencing factors such as their respiration rate, the speed of photosynthetic enzyme catalysis, and nutrient absorption [56,57]. Notably, although phytoplankton abundance is relatively high in summer, the species number is relatively low. This may be due to the increased abundance of Cyanophyta during the summer months, leading to decreased species diversity. Cyanophyta thrives in high-temperature and high-light conditions [58]. Therefore, as temperature increases starting in April, the abundance of Cyanophyta begins to rise, reaching 0.31 × 10^6^ ind. L^−1^ (comprising 18.0% of the total) then peaking in summer at 1.03 × 10^6^ ind. L^−1^ (59.1%). Meanwhile, the calculated dominance index (Y) indicated that the dominant species in the LYR during the summer is *Pseudanabaena mucicola* (Y = 4405), whereas in the other three seasons, it is primarily Bacillariophyta. It is understood that *Pseudanabaena mucicola* is one of the common Cyanophyta in wastewater treatment, which produces algal toxins and 2-methylisoborneol [59]. Algal toxins, including hepatotoxins and neurotoxins, can harm the growth and development of aquatic organisms [60]. 2-methylisoborneol produces an unpleasant odor when its concentration exceeds 10 ng L^−1^, posing a threat to drinking water supply safety [59]. Consequently, this study emphasizes the importance of strengthening monitoring efforts for Cyanophyta, particularly *Pseudanabaena mucicola*. Interestingly, the phytoplankton abundance at site S18 was the highest, reaching 6.1 × 10^6^ ind. L^−1^, which is 3.5–17.7 times that of other sections (Fig. 4b). This area can only be reached by government sampling boats, and few travelers visit there. In this case, the high phytoplankton abundance at site S18 may be related to the low level of human activity at that location. In addition, the slow flow rate caused by a wider river channel may also be one of the reasons.

In addition to phytoplankton, this study identified 55 species of zooplankton, which comprised 31 Rotifera species (accounting for 56.4% of the total species), 16 Copepoda species (29.1%), and eight species of Cladocera (14.5%). Interestingly, the species number observed in summer (23 species) was significantly lower than that recorded in the other three seasons, namely autumn (45 species), spring (39 species), and winter (39 species).

The annual average abundance of zooplankton in the LYR was 182.21 ind. L^−1^. Among the zooplankton, Rotifera had the highest abundance, with 112.05 ind. L^−1^, accounting for 61.5% of the total abundance. Copepoda followed with an abundance of 49.71 ind. L^−1^, constituting 27.3% of the total, and Cladocera had an abundance of 20.45 ind. L^−1^, making up 11.2% of the total. The composition of zooplankton species and their abundance indicated a trend of miniaturization of zooplankton communities. This could be related to the preference of filter-feeding fish for large planktonic animals such as Copepoda and Cladocera. Silver carp and Bighead, as important economic fish species in the LYR, feed on a large quantity of large planktonic animals. This predation pressure could contribute to the dominance of Rotifera in the water [61]. This trend will lead to a decrease in plankton diversity, thereby affecting the stability of the ecosystem, which is worth paying attention to Ref. [62].

The spatiotemporal distribution of zooplankton abundance is shown in Fig. 4c and d. From the perspective of the time factor, there were significant differences in zooplankton abundance among different seasons (*p* < 0.05), with an overall pattern of summer (385.23 ind. L^−1^) > spring (182.18 ind. L^−1^) > autumn (133.83 ind. L^−1^) > winter (27.61 ind. L^−1^). Particularly, the abundance of Rotifera in summer reached 366.48 ind. L^−1^ (accounting for 95.1%), 8.4–13.3 times higher than in other seasons. Upon comparing the composition of phytoplankton abundance, it was observed that Rotifera abundance and Cyanophyta abundance exhibited similar seasonal trends. This can be partially attributed to bottom-up effects and the direct influence of WT on the growth and reproduction of phytoplankton and zooplankton [63]. When cyanobacterial blooms occurred in the river, the size of Cladocera individuals decreased significantly, weakening their competitive inhibition on Rotifera. This indirectly led to an increase in the dominance of Rotifera [62]. Furthermore, the calculation of the dominance index demonstrated that the dominant species of zooplankton in the LYR exhibited seasonal succession. Keratella cochlearis was the first dominant species in both summer (Y = 0.5386) and winter (Y = 0.4223), whereas, in spring and autumn, Copepoda was the dominant species. This seasonal variation may be associated with the prolific reproduction of Cyanophyta. Previous studies have indicated that Keratella cochlearis is less affected by Cyanophyta [62].

When considering spatial distribution, the overall trend revealed a gradual decrease in zooplankton abundance from upstream to downstream: upstream (276.47 ind. L^−1^) > midstream (179.54 ind. L^−1^) > downstream (92.40 ind. L^−1^). This spatial pattern could be influenced by variations in water quality across different regions.

### 3.4 Multi-dimensional water's ecological health assessment

**Fig. 5:**
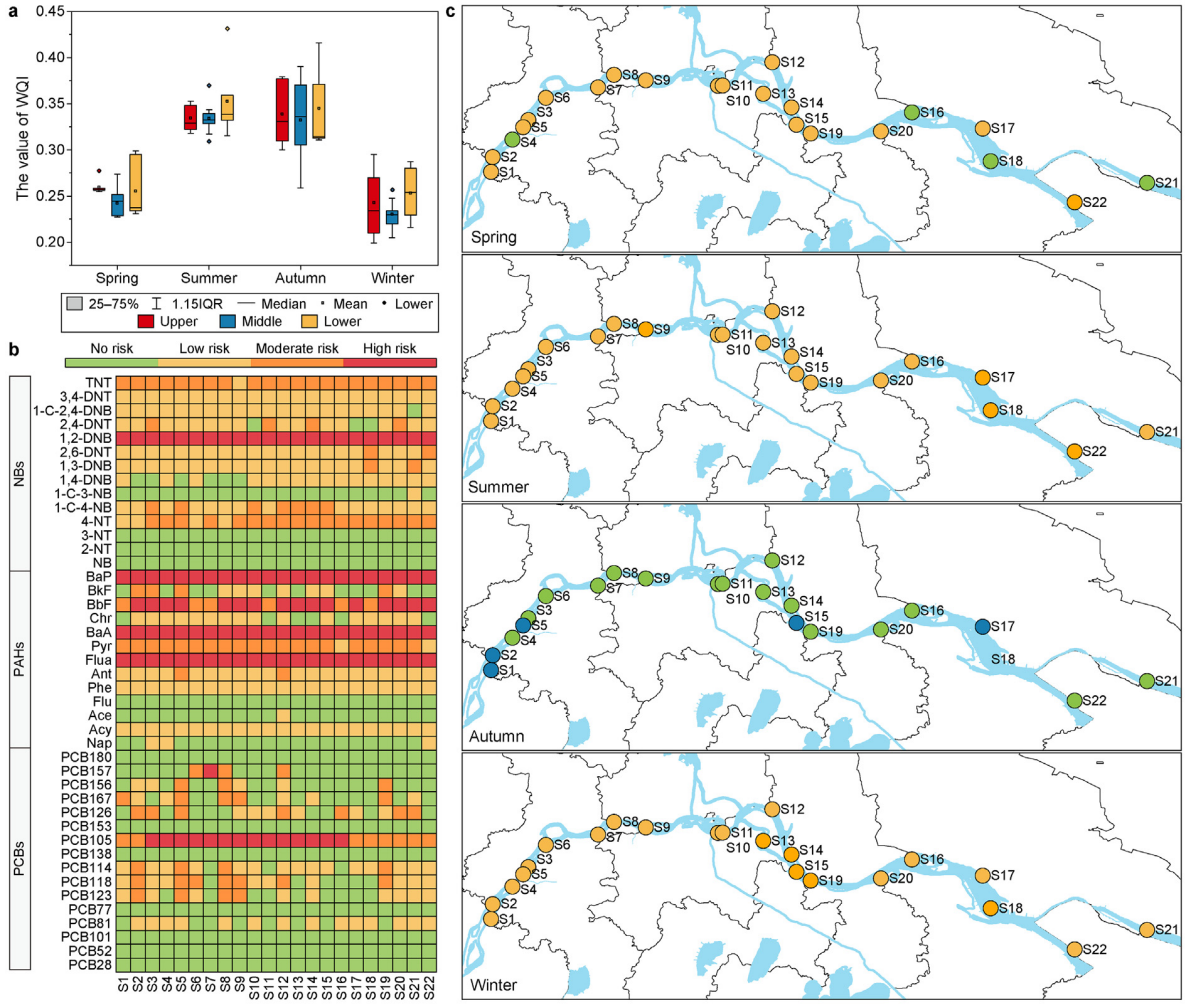
Distribution of evaluation results for three methods. **a**, The spatiotemporal distribution of WQI; **b**, Annual average RQ value of PCBs, PAHs, and NBs; **c**, The spatiotemporal distribution of P-IBI health level.

The distribution of WT, pH, DO, COD_Mn_, TP, TN, NH_3_-N, and NO_3_-N in the LYR is illustrated in Fig. S6. DO, COD_Mn_, NH_3_-N, and TP met the Class II water quality standards as per the Environmental Quality Standards for Surface Water (GB 3838-2002). The specific standard limits of various pollutants are shown in Table S13. However, among all conventional indicators, TN pollution is relatively severe, with 11.5% of the samples falling into Class IV and 83.9% into Class V. As shown in Fig. 5a, the WQI values across the LYR ranged from 0.20 to 0.43, with an average value of 0.29. The grading evaluation results revealed that 66.7% of the samples were relatively clean, while the rest were categorized as clean. Regarding temporal variations, the WQI in spring and winter was significantly lower than that in summer and autumn (*p* < 0.05), indicating that the water quality of the surface water of the LYR was comparatively better in spring and winter. Concerning spatial variations, there were minimal differences in the annual mean WQI at each sampling site. In summary, although the conventional water quality pollution in the LYR is relatively light at present, TN pollution cannot be ignored.

In contrast to the findings from the WQI evaluation, the results of the ecological risk assessment for the 43 high-risk emerging contaminants manifested that six compounds, specifically PCB105, fluoranthene (Flua), benzo(*a*)anthracene (BaA), Benzo(*b*)fluorathene (BbF), benzo(*a*)pyrene (Bap), and 1,2-dinitrobenzene (1,2-DNB), posed high environmental risks at over 50% of the sampling sites (Fig. 5b). The RQ value for PCB105 ranged from 0.42 to 1.81, signifying high risk at 63.6% of the sampling points and moderate risk at the remaining sites. Notably, four of the six high-risk substances belong to PAHs, and PAHs contributed the most to the total ecological risk, accounting for 81–93%. Specifically, Flua, BaA, and Bap all exhibited a high-risk status at 100% of the sites, while BbF had high risk at 72.7% of the sites and moderate risk at 27.3%. These four compounds belong to high molecular weight (HMW) PAHs and have higher lipophilicity and toxicity [64]. Bap has been recognized as a Class I carcinogen by the International Agency for Research on Cancer of the World Health Organization. Studies have shown that long-term exposure to low-dose Bap can induce various cancers [65]. Therefore, the government should focus on the ecological risks of HMW PAHs. For NBs, only 1,2-DNB posed a high risk to the environment. These results underscore the importance of considering both the concentrations and toxicities of pollutants when assessing their ecological impact on the water environment. While the concentrations of most NBs were higher than those of PAHs, PAHs predominantly contribute to the environmental risk in the surface water of the LYR, emphasizing the need for heightened attention in their risk management and control. Apart from ecological risk assessment, it is necessary to analyze human health risk assessments further in the future.

The RQ values exhibited varying distribution patterns across different seasons due to fluctuations in pollutant concentrations. In the summer, the environmental risk peaked, with the RQ values ranging from 0 to 83.7 (Figs. S7–S10). This period saw four high-risk and ten medium-risk substances, with BaA making the most substantial contribution to the ecological risk. Conversely, spring had the lowest ecological risk, with only Flua and 1,2-DNB classified as high-risk substances. The wet season generally represented a high-risk period for the pollution of PCBs, PAHs, and NBs in the LYR. This heightened risk during the wet season was primarily attributed to increased inputs of exogenous pollutants. Therefore, strengthening the control of exogenous organic pollutants is crucial. Regarding spatial distribution, sampling site S19 had the highest comprehensive ecological risk with a total RQ (∑_43_RQ) of 66.2, while site S1 exhibited the lowest value of 26.5.

The P-IBI results provided insights into the health of the aquatic ecosystem. The distribution of water's ecological health levels based on P-IBI assessments showed that the proportion of samples falling into “excellent”, “good”, “fair”, and “poor” health grades was 5.4%, 23.0%, 59.7%, and 11.9%, respectively. When considering temporal variations, the integrity of aquatic organisms in autumn was prominently superior to the other three seasons (*p* < 0.01). In autumn, 81.0% of samples were classified as “good”, with 19.0% classified as “excellent”. In contrast, during spring, winter, and summer, the proportion of samples categorized as “fair” was 77.3%, 68.2%, and 59.1%, respectively. These seasonal differences in biological integrity can be attributed in part to the selected evaluation indicators, as the value of P-IBI results from the combined effect of multiple elements [66]. The lower plankton integrity observed in summer and winter may be linked to prominent seasonal variations in indicators such as M18 (Copepoda abundance), M27 (Relative abundance of the top three dominant species of phytoplankton), M32 (Relative abundance of Rotifera), M36 (The Shannon-Wiener index of Phytoplankton), and M39 (The Shannon-Wiener index of zooplankton) (Fig. S11). This reminds us to pay more attention to the abundance changes of dominant species of phytoplankton, Rotifera, and Copepoda in zooplankton.

Considering spatial variations, it was observed that the biological integrity was slightly better in the upstream areas compared to the midstream and downstream regions. However, the differences were not highly significant. For the other 21 sites, the average annual P-IBI rating was categorized as “fair”, except for site S2, which rated as “good”. The scores of M14 (the number of Copepoda species) and M39 (Zooplankton biodiversity index) at site S2 were significantly higher than those at other sites. This indicated that the health level at site S2 was distinguished from other sites mainly because of the better zooplankton diversity.

### 3.5 Comparative analysis of evaluation results of three methods

To compare three different evaluation results, the values of WQI and RQ were standardized based on the scoring methodology employed in P-IBI. The classification criteria for health grades were consistent with those used in P-IBI. The correlation analysis revealed no significant correlation between the evaluation results of WQI, RQ, and P-IBI, indicating that variation in water quality may not necessarily determine the integrity of aquatic organisms. Wu et al. [67] found that the WQI and P-IBI evaluation results of Taihu Lake were opposite, which was consistent with the conclusion of this study. Therefore, when conducting water ecological research, we cannot rely solely on chemical indicators, especially conventional water quality, to judge water quality. We should evaluate the health status of water ecology from multiple dimensions, including chemistry, biology, and even humanities.

**Fig. 6:**
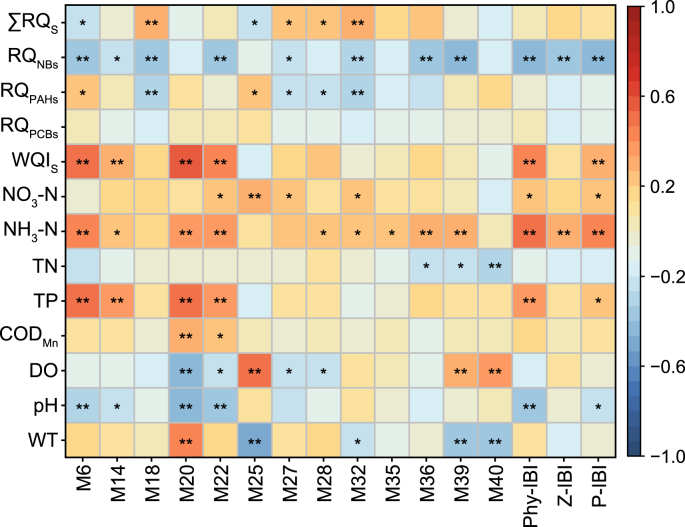
Correlation analysis between water quality and biological indicators. In the figure, the ∑RQs and WQIs were standardized values, and Z-IBI represented zooplankton-IBI (∗*p* < 0.05, ∗∗*p* < 0.01).

As shown in Fig. 6, although WQI was not correlated with P-IBI, it did show significant correlations with biological indicators such as M6, M14, M20, and M22 (*p* < 0.05). Similarly, despite no obvious correlation between RQ and P-IBI, RQ was significantly correlated with M6, M18, M25, M27, M28, and M32. Hu et al. [68] also found no direct correlation between WQI and P-IBI results, but some water quality parameters were highly correlated with P-IBI evaluation results and certain biological indicators. Additionally, when examining the correlation between individual water quality parameters and the IBI system, six conventional water quality parameters, except for COD_Mn_ and TN, exhibited significant correlations with most biological indicators. TP and NH_3_-N strongly correlated with four and nine biological indicators, respectively. TP had a negative correlation with both Phy-IBI and P-IBI, while NH_3_-N exhibited negative correlations with all three IBI evaluation results, indicating that increased nutrient concentrations could harm the ecological health of the water. This well agrees with the study of Welch et al. [69]. Regarding high-risk emerging contaminants, there was no apparent correlation between RQ_PCBs_ (the RQ of PCBs) and biological indicators, which could be attributed to the primary source of PCBs. On the other hand, RQ_PAHs_ (the RQ of PAHs) showed significant correlations with six biological indicators (*p* < 0.05). PAHs can bind to phytoplankton or debris and accumulate in zooplankton through the food chain [70]. NBs exhibited significant negative correlations with most of the biological indicators (*p* < 0.05), which could be related to the pronounced seasonal variation trend of NBs. To further explore the impact of environmental factors on plankton communities, we conducted RDA and CCA analyses. The results showed that the total explanatory rates of 11 environmental variables for phytoplankton and zooplankton were 55.4% and 44.2%, respectively, indicating that environmental factors cannot fully explain the changes in plankton communities (Fig. S12). Among them, the main environmental factors affecting phytoplankton were DO and WT, while the main environmental factor affecting zooplankton was WT. The response of different groups to environmental factors is different, indicating the necessity to select a variety of biological groups to evaluate the water's ecological health [24].

In summary, it's important to recognize that water quality parameters can indirectly influence the results of P-IBI evaluation by impacting the plankton community. This impact is attributable not solely to the water quality parameters outlined in this study but also to the comprehensive effects of river hydrological conditions, lighting conditions, climate change, and other aquatic organisms [27,71]. Therefore, the P-IBI evaluation approach is valuable as it effectively captures the current state of regional water's ecological health and complements the limitations of chemical-based evaluations alone. This holistic assessment considers the broader ecosystem and its biological components, providing a more comprehensive understanding of water quality and ecological integrity.

However, due to time and experimental conditions limitations, this paper considered only eight conventional water quality indices, 43 high-risk emerging contaminants, and phytoplankton, and it ignored the effects of other pollutants and aquatic organisms. Moreover, this study only compared the three evaluation methods of WQI, RQ, and P-IBI. In the future, the entropy method, principal component analysis, fuzzy comprehensive evaluation method, and other methods can be considered to determine the contribution of each part.

## 4 Conclusions

(1) High-risk emerging contaminants: All 43 high-risk emerging contaminants were detected in the LYR, with concentrations ranging from 173.19 to 3853.46 ng L^−1^. NBs were the primary contributors, making up 25.0–89.7% of the total, followed by PAHs (10.2–71.6%) and PCBs (0.1–3.4%). Notably, these compounds were more prevalent and concentrated in summer. Spatial analysis revealed that the highest concentrations were found downstream, with decreasing levels observed in midstream and upstream locations.
(2) Phytoplankton and zooplankton: The LYR contained 81 species of phytoplankton and 55 species of zooplankton. Bacillariophyta had the highest species count and abundance. During summer, there was a significant increase in Cyanophyta, particularly *Pseudanabaena mucicola* (Y = 0.4405), which reduced species diversity. Zooplankton communities, with the highest species number and abundance of Rotifera, trended toward miniaturization, potentially due to predation by filter-feeding fish.
(3) Water quality: In the LYR, Conventional water quality indices generally indicated compliance with Class II water quality criteria, reflecting relatively good water conditions per the WQI. However, a different picture emerged when assessing RQs, with six compounds posing high environmental risks, mainly PAHs. P-IBI evaluations revealed that the ecological health of most LYR water samples (59.7%) was rated as “fair”, highlighting the potential for enhancement.
(4) Correlations: Certain water quality parameters were highly correlated with the results of P-IBI and biological indicators. This suggests that water quality parameters can indirectly influence P-IBI results by impacting the plankton community. While the three evaluation methods showed differences, the LYR's ecological health, especially concerning high-risk substances and ecological integrity, requires further attention and restoration efforts.

## CRediT authorship contribution statement

**Qiuyun Zhao:** Writing - Original Draft, Conceptualization, Data Curation, Methodology, Visualization. **Yangyang Zhang:** Investigation, Methodology, Conceptualization, Formal Analysis. **Xiuwen Li:** Visualization, Writing - Review & Editing, Formal Analysis, Software, Supervision. **Xiaodong Hu:** Investigation, Software, Validation. **Rui Huang:** Software, Investigation, Validation. **Jixiong Xu:** Investigation, Software, Validation. **Zilong Yin:** Investigation, Software, Validation. **Xinjie Gu:** Investigation. **Yuncheng Xu:** Investigation. **Jinbao Yin:** Writing - Review & Editing. **Qing Zhou:** Writing - Review & Editing. **Aimin Li:** Writing - Review & Editing. **Peng Shi:** Funding Acquisition, Project Administration, Supervision.

## Declaration of competing interest

The authors declare that they have no known competing financial interests or personal relationships that could have appeared to influence the work reported in this paper.
