# Supplementary material for: Evaluating a river's ecological health: A multidimensional approach
Source: Environ Sci Ecotechnol. 2024 Apr 18;21:100423. doi: 10.1016/j.ese.2024.100423 (PMC11061703; doi:10.1016/j.ese.2024.100423)
Supplement: Multimedia component 1 [file mmc1.docx]

Supporting Information for

**“**Evaluating a river's ecological health: A multidimensional approach**”**

**Authors:**

Qiuyun Zhao^a,#^, Yangyang Zhang^a,#^, Xiuwen Li^a*^, Xiaodong Hu^b^, Rui Huang^b^, Jixiong Xu^b^, Zilong Yin^b^, Xinjie Gu^a^, Yuncheng Xu^a^, Jinbao Yin^a^, Qing Zhou^a^, Aimin Li^a^, Peng Shi^a*^

**Affiliations of authors:**

^a^ State Key Laboratory of Pollution Control and Resource Reuse, School of the Environment, Nanjing University, Nanjing 210023, China.

^b^ Jiangsu Hydraulic Research Institute, Nanjing 210023, China.

^#^**Co-first author**

***Corresponding author**

**Addresses**:

State Key Laboratory of Pollution Control and Resource Reuse, School of the Environment, Nanjing University, 163 Xianlin Avenue, Nanjing 210023, China.

**Phone:** +86-25-89680508;

**Fax:** +86-25-89680508;

**Email:** lixiuwen@nju.edu.cn (Xiuwen Li); shipeng@nju.edu.cn (Peng Shi).

**Total Pages: 31**

**Number of texts: 5**

**Number of tables: 14**

**Number of figures: 6**

**Contents**

**Text S1.** Pretreatment for high-risk emerging contaminants.

**Text S2.** Quality assurance and quality control of high-risk emerging contaminants.

**Text S3.** The detailed calculation formula of Y.

**Text S4.** The calculation formula for WQI.

**Text S5.** The calculation process for RQ.

**Text S6.** The screening steps for alternative indicators.

**Text S7.** The calculation process for P-IBI.

**Table S1.** Detailed information about the reagents.

**Table S2.** The information of sampling locations in the LYR.

**Table S3.** GC/MS parameters of 3 types of high-risk emerging contaminants.

**Table S4.** Retention time, quantitative ions and qualitative ions of 43 high-risk emerging contaminants.

**Table S5.** Recovery rate and method detection limit (MDL) of PCBs.

**Table S6.** Recovery rate and method detection limit (MDL) of PAHs.

**Table S7.** Recovery rate and method detection limit (MDL) of NBs.

**Table S8.** Acute and chronic toxicity data and PNEC for 43 compounds.

**Table S9.** 41 alternative indicators of plankton and their interference response.

**Table S10.** Correlation analysis results of 23 alternative indicators in the LYR.

**Table S11.** Calculation standard of ratio method for core indicators.

**Table S12.** Summary of reported PCBs, PAHs, and NBs concentrations in surface water around the world.

**Fig. S1.** The boxplot for the discriminant ability test of 31 alternative indicators in the LYR.

**Fig. S2.** Concentration distribution of PCBs, PAHs, and NBs in the LYR.

**Fig. S3.** Cumulative bar chart of the concentrations of 43 compounds in all seasons. The data for each season are divided into three categories, including PCBs, PAHs, and NBs.

**Fig. S4.** Cross plots for the isomeric ratios of Fla/ (Fla + Pyr) versus Ant / (Ant + Phe) (a), and Fla/ (Fla + Pyr) versus BaA / (BaA + Chr) (b).

**Fig. S5.** Seasonal distribution of conventional index (a: WT, b: pH, c: DO, d: COD_Mn_, e: TP, f: TN, g: NH_3_-N, h: NO_3_-N) in the LYR.

**Fig. S6.** RQ value of PCBs, PAHs, and NBs in Spring in the LYR.

**Fig. S7.** RQ value of PCBs, PAHs, and NBs in Summer in the LYR.

**Fig. S8.** RQ value of PCBs, PAHs, and NBs in Autumn in the LYR.

**Fig. S9.** RQ value of PCBs, PAHs, and NBs in Winter in the LYR.

**Fig. S10.** Seasonal distribution of 13 core Index scores.

**Text S1.** Pretreatment steps for high-risk emerging contaminants.

Firstly, the water samples were passed through 0.45 μm glass-fiber filter (Millipore, U.S.) by vacuum suction filtration device, and were adjusted pH < 3 with hydrochloric acid. Then 1 L of the treated water sample was extracted for SVOCs using the automatic solid-phase extraction instrument (SPE, Thermo Scientific, Autotrace 280, U.S.), with Waters Oasis HLB cartridge (Waters, 500 mg/6 mL, Milford, MA, U.S.). The solid phase extraction procedure is as follows: The cartridge was activated by 10 mL methanol and ultrapure water, respectively. Next, 1 L water sample was loaded at the rate of 10 mL/min. Then the cartridge was dried with high-purity nitrogen for 20 min. Subsequently, the cartridge was eluted with 5 mL dichloromethane, ethyl acetate, hexane, and methanol, respectively twice. After the extraction, the eluent was concentrated by a rotary evaporation instrument (IKA, RV8 HB10, Germany) and a dry nitrogen blower (Ningbo Licheng, MD200-1N, China) at 30 °C, and then the concentrate was fixed to 1 mL with hexane, at which time the concentration factor of the sample was 1000. Eventually, the samples were filtered with 0.22 μm Organic membrane filter (PALL, 0.2 μm, U.S.) and transferred to vial, and then stored at −20 °C until testing.

**Text S2.** Quality assurance and quality control of high-risk emerging contaminants.

The standard curves of 7 concentration gradients (PCBs: 0.5–50 μg/L, PAHs and NBs: 5–500 μg/L) were established, and the correlation coefficients of each substance were satisfied with R2 > 0.99. To ensure the sensitivity and accuracy of the instrument, one injection of the solvent blank was added for every ten samples. Simulated water samples at concentrations of 0.5, 5, and 20 ng/L were prepared, with 7 parallels for each concentration. After the same extraction concentration, and detection steps as the samples, the limit of detection (MDL) was obtained by calculating the standard deviation between the 7 parallel samples and multiplying by 3.14. The MDL of PCBs, PAHs, and NBs were 0.12–0.39 ng/L, 0.40–3.28 ng/L, and 2.66–22.74 ng/L, respectively. Then, the low, medium and high concentrations of simulated water samples were prepared respectively, with three parallels for each concentration. After extraction and detection, the recovery rate was calculated. The recoveries of all target substances ranged from 62.8% to 114.1%.

**Text S3.** The detailed calculation formula of Y.

The dominance index (Y) is used to determine the dominance of plankton. A dominant species is defined when Y ≥ 0.02. The calculation formula is as follows:

$$Y=f_{i}\times\frac{n_{i}}{N}$$

Where, N represents the total abundance of all species (ind/L), n_i_ represents the abundance of species i (ind/L), and f_i_ represents the occurrence frequency of that species.

**Text S4.** The calculation formula for WQI.

$WQI=\frac{\sum_{i=1}^{n} P_{i}}{n}$ (1)

In the formula, P_i_ is the single pollution index of the i-th indicator, and n is the total number of conventional water quality indexes. The lower the values of COD_Mn_, TP, TN, NH_3_-N, and NO_3_-N, the better the water quality, so its P_i_ is the ratio of the measured value of the i-th indicator to the standard value. Inversely, the P_i_ of DO is the ratio of the standard value of the i-th indicator to the measured value. The standard value is refered to Environmental Quality Standard for Surface Water in China.

**Text S5.** The calculation process for RQ.

The calculation formula is as follows:

$RQ=\frac{\mathrm{MEC}}{\mathrm{PNEC}}=\frac{\mathrm{MEC}}{{NOEC or E{\left( L \right)C}_{50}}/\mathrm{AF}}$ (2)

Where, MEC is the actual concentration of compounds measured in this study, and PNEC is deduced according to the assessment factor (AF) provided by the European Union Risk Assessment Technical Guidelines. Acute or chronic toxicity data was obtained through the EPA ECOTOX database (https://cfpub.epa.gov/ecotox/). When one, two, and three trophic levels (large algae, fleas, and fish) of chronic toxicity data are available, AF are 100, 50, and 10, respectively, and When only acute toxicity data were available, AF is 1000. Furthermore, when toxicity data were lacking, T.E.S.T 5.1.2 software was used to simulate and calculate the acute EC_50_ data of different compounds for the two trophic levels of fleas and fish. The lowest EC_50_ was selected to reflect the worst-case scenario, while the corresponding AF was 1000.

**Text S6.** The screening steps for alternative indicators.

The screening steps for the indicators were as follows: (a) Distribution range test: the detection rate of each indicator was calculated to eliminate indicators with undetected rates exceeding 95%. (b) Discrimination ability test: the overlap degree of box IQ between reference points and impaired points was compared to select indicators with IQ ≥ 2 for further analysis. (c) Correlation analysis: Using the Pearson method to verify the independence of each parameter to prevent information redundancy. If the correlation coefficient |*r*| > 0.75, then one of the two indicators should be excluded.

**Text S7.** The calculation process for P-IBI.

Due to dimensional differences between different parameters, the ratio method was used to unify the dimensions. When the value of the indicator decreases or increases with increasing interference, its calculation formula is respectively Equation (3) or Equation (4).

$S=P/P_{95\%}$ (3)

$S=(P_{\mathrm{MAX}}-P)/(P_{\mathrm{MAX}}-P_{5\%})$ (4)

Where P is the measured value of each indicator; P_95%_ is the 95% quantile of the index, that is, the best expected value; P_5%_ is the 5% quantile of the index; P_MAX_ is the maximum value of this indicator. If the S value is greater than 1, record as 1.

The IBI value of each point is equal to the sum of the S values of all core indicators at that point, that is

$P-IBI'=\sum_{i=1}^{n} S_{i}$ (5)

Where S_i_ is the S value of the i-th core indicator, and n is the total number of core indicators.

Then, the P-IBI' value was standardized by calculating Equation (6):

$P-IBI=\frac{P-IBI'}{{P-IBI'}_{95\%}}$ (6)

**Table S1.** Detailed information about the reagents.

| **Name of reagents and consumables** | **Purity/Specification** | **Manufacturers** |
| --- | --- | --- |
| Hydrochloric acid | Guaranteed reagent | Sinopharm Chemical Reagent Co. Ltd |
| methanol | chromatographically pure | Sigma-Aldrich |
| Ethyl acetate | chromatographically pure | Sigma-Aldrich |
| Methylene chloride | chromatographically pure | Sigma-Aldrich |
| n-hexane | chromatographically pure | Sigma-Aldrich |
| 18 PCBs mixed standard | >98% | o2si |
| 16 PAHs mixed standard | >98% | o2si |
| 15 NBs mixed standard | >98% | o2si |
| HLB solid phase extraction column | 500mg/6mL | Waters |
| High purity nitrogen | >99.999% | Chuangda special gas |
| Organic membrane filter | 0.22μm | PALL |
| Drainage membrane filter | 0.45μm | Jin teng |
| Lugol’s solution | 5% (V/V) | Shanghai yuanye Bio-Technology Co. Ltd |
| Formaldehyde solution | w (HCHO)＝37–40% | Aladdin |

**Table S2.** The information of sampling locations in the lower reaches of the Yangtze River.

| **Number** | **Sampling locations** | **Latitude(ºE)** | **Longitude (ºN)** |
| --- | --- | --- | --- |
| S1 | Nanjing - Anhui (South) | 118.50471 | 31.78033 |
| S2 | Nanjing - Anhui (North) | 118.51229 | 31.85234 |
| S3 | National aquatic germplasm resources protection zone | 118.68262 | 32.02852 |
| S4 | Zimu Island, Nanjing | 118.6067301 | 31.93523372 |
| S5 | Jiangxin Island, Nanjing | 118.6588243 | 31.99399972 |
| S6 | Bagua Island, Nanjing | 118.7664722 | 32.13492181 |
| S7 | Longtan Jiangxin Island, Nanjing | 119.016326 | 32.18489 |
| S8 | Nanjing - Yangzhou | 119.09282 | 32.24489 |
| S9 | Nanjing - Zhenjiang | 119.244562 | 32.2182 |
| S10 | Jiangxin Island, Zhenjiang 1 | 119.588872 | 32.192613 |
| S11 | Jiangxin Island, Zhenjiang 2 | 119.611723 | 32.193813 |
| S12 | Yangzhou - Taizhou | 119.849183 | 32.305278 |
| S13 | Yangtze River Knife Crucian carp Reserve | 119.805184 | 32.155082 |
| S14 | Jiangxin Island, Taizhou | 119.94 | 32.088761 |
| S15 | Zhenjiang - Changzhou | 119.96416 | 32.00704 |
| S16 | Taizhou - Nantong | 120.51601 | 32.06494 |
| S17 | Nantong Yao Gang | 120.8546 | 31.988705 |
| S18 | Suzhou iron yellow sand | 120.892635 | 31.832164 |
| S19 | Changzhou - Wuxi | 120.031563 | 31.963864 |
| S20 | Wuxi - Suzhou | 120.36826 | 31.97547 |
| S21 | Nantong downstream | 121.63889 | 31.72845 |
| S22 | Suzhou downstream | 121.2927647 | 31.63517183 |

**Table S3.** GC/MS parameters of 3 types of high-risk emerging contaminants.

| **Compound** | **Gas chromatographic conditions** |
| --- | --- |
| PCBs | Heating procedure: the initial temperature is 120 ℃ (1 min), at the rate of 20 ℃/min to 180 ℃, then at the rate of 5 ℃/min to 280 ℃. Split injection, split ratio of 10:1. The column flow rate was 1.0 mL/min with He as carrier gas. |
| PAHs | Heating procedure: the initial temperature is 60 ℃(1 min), at the rate of 20℃/min to 180℃(5min), then at the rate of 10 ℃/min to 290 ℃. Split injection, split ratio of 10:1. The column flow rate was 1.0 mL/min with He as carrier gas. |
| NBs | Heating procedure: the initial temperature is 60 ℃, at the rate of 10 ℃/min to 200 ℃, then at the rate of 15 ℃/min to 250 ℃. Split injection, split ratio of 10:1. The column flow rate was 1.0 mL/min with He as carrier gas. |

**Table S4.** Retention time (*R*t), quantitative ions and qualitative ions of 43 high-risk emerging contaminants.

| **Compound** | **Abbreviation** | **CAS No.** | **Rt (min)** | **Quantitative ion** | **Qualitative ion** |
| --- | --- | --- | --- | --- | --- |
| PCB28 | PCB28 | 7012-37-5 | 9.59 | 258 | 256 |
| PCB52 | PCB52 | 35693-99-3 | 10.52 | 292 | 290 |
| PCB101 | PCB101 | 37680-73-2 | 13.09 | 326 | 328 |
| PCB81 | PCB81 | 70362-50-4 | 13.96 | 292 | 290 |
| PCB77 | PCB77 | 32598-13-3 | 14.29 | 292 | 290 |
| PCB123 | PCB123 | 65510-44-3 | 14.97 | 326 | 324 |
| PCB118 | PCB118 | 31508-00-6 | 15.09 | 326 | 324 |
| PCB114 | PCB114 | 74472-37-0 | 15.41 | 326 | 324 |
| PCB138 | PCB138 | 35065-28-2 | 15.79 | 360 | 362 |
| PCB105 | PCB105 | 32598-14-4 | 15.90 | 326 | 324 |
| PCB153 | PCB153 | 35065-27-1 | 16.67 | 360 | 362 |
| PCB126 | PCB126 | 57465-28-8 | 17.03 | 326 | 324 |
| PCB167 | PCB167 | 52663-72-6 | 17.64 | 360 | 362 |
| PCB156 | PCB156 | 38380-08-4 | 18.38 | 360 | 362 |
| PCB157 | PCB157 | 69782-90-7 | 18.53 | 360 | 362 |
| PCB180 | PCB180 | 35065-29-3 | 18.92 | 394 | 396 |
| Naphthaline | Nap | 91-20-3 | 6.00 | 128 | 127 |
| Acenaphthylene | Acy | 208-96-8 | 8.09 | 152 | 151 |
| Acenaphthene | Ace | 83-32-9 | 8.40 | 153 | 152 |
| Fluorene | Flu | 86-73-7 | 9.54 | 166 | 165 |
| Phenanthrene | Phe | 85-01-8 | 12.99 | 178 | 176 |
| Anthracene | Ant | 120-12-7 | 13.19 | 178 | 176 |
| Fluoranthene | Flua | 206-44-0 | 17.14 | 202 | 200 |
| Pyrene | Pyr | 129-00-0 | 17.79 | 202 | 200 |
| Benzo(a)anthracene | BaA | 56-55-3 | 21.17 | 228 | 226 |
| Chrysene | Chr | 218-01-9 | 21.26 | 228 | 226 |
| Benzo[b]fluorathene | BbF | 205-99-2 | 23.80 | 252 | 250 |
| Benzo[k]fluorathene | BkF | 207-08-9 | 23.88 | 252 | 250 |
| Benzo(a)pyrene | Bap | 50-32-8 | 24.66 | 252 | 250 |
| Nitrobenzene | NB | 98-95-3 | 6.09 | 77 | 123 |
| 2-Nitrotoluene | 2-NT | 88-72-2 | 7.11 | 120 | 65 |
| 3-Nitrotoluene | 3-NT | 99-08-1 | 7.61 | 91 | 137 |
| 4-Nitrotoluene | 4-NT | 99-99-0 | 7.92 | 91 | 137 |
| 1-Chloro-3-Nitrobenzene | 1-C-3-NB | 121-73-3 | 8.08 | 111 | 75 |
| 1-Chloro-2-Nitrobenzene | 1-C-2-NB | 88-73-3 | 8.28 | 111 | 75 |
| p-Dinitrobenzene | 1,4-DNB | 100-25-4 | 10.83 | 168 | 75 |
| m-Dinitrobezene | 1,3-DNB | 99-65-0 | 10.99 | 76 | 168 |
| 2,6-Dinitrotoluene | 2,6-DNT | 606-20-2 | 11.08 | 165 | 89 |
| 1,2-Dinitrobenzene | 1,2-DNB | 528-29-0 | 11.17 | 168 | 50 |
| 2,4-Dinitrotoluene | 2,4-DNT | 121-14-2 | 12.02 | 165 | 89 |
| 1-chloro-2,4-dinitrobenzene | 1-C-2,4-DNB | 97-00-7 | 12.38 | 202 | 75 |
| 3,4-Dinitrotoluene | 3,4-DNT | 610-39-9 | 12.63 | 182 | 63 |
| Trinitrotoluene | TNT | 118-96-7 | 13.91 | 210 | 89 |

**Table S5.** Recovery rate and method detection limit (MDL) of PCBs.

| **Compound** | **Recovery rate (%)** | | | **MDL (ng/L)** |
| --- | --- | --- | --- | --- |
|  | **2 ng/L** | **10 ng/L** | **20 ng/L** |  |
| PCB28 | 71.4 | 81.9 | 78.7 | 0.21 |
| PCB52 | 76.5 | 83.2 | 84.4 | 0.25 |
| PCB101 | 65.7 | 70.9 | 76.6 | 0.29 |
| PCB81 | 83.3 | 75.9 | 78.0 | 0.11 |
| PCB77 | 90.5 | 86.8 | 97.2 | 0.40 |
| PCB123 | 79.3 | 78.1 | 71.6 | 0.32 |
| PCB118 | 72.9 | 77.6 | 74.8 | 0.34 |
| PCB114 | 71.5 | 78.6 | 76.1 | 0.15 |
| PCB138 | 78.5 | 87.8 | 62.8 | 0.12 |
| PCB105 | 98.0 | 94.1 | 108.9 | 0.37 |
| PCB153 | 72.1 | 68.9 | 76.5 | 0.39 |
| PCB126 | 86.6 | 87.6 | 89.0 | 0.25 |
| PCB167 | 81.2 | 72.0 | 77.2 | 0.27 |
| PCB156 | 83.9 | 92.0 | 87.3 | 0.22 |
| PCB157 | 76.4 | 74.4 | 80.5 | 0.18 |
| PCB180 | 77.5 | 78.8 | 73.0 | 0.12 |

**Table S6.** Recovery rate and method detection limit (MDL) of PAHs.

| **Compound** | **Recovery rate (%)** | | | **MDL (ng/L)** |
| --- | --- | --- | --- | --- |
|  | **20 ng/L** | **100 ng/L** | **200 ng/L** |  |
| Nap | 72.8 | 82.0 | 76.5 | 1.12 |
| Acy | 88.0 | 92.1 | 90.3 | 0.40 |
| Ace | 94.1 | 99.4 | 80.1 | 0.87 |
| Flu | 93.0 | 92.6 | 95.2 | 1.02 |
| Phe | 88.4 | 84.8 | 113.3 | 3.28 |
| Ant | 83.6 | 92.4 | 95.7 | 1.16 |
| Flua | 90.1 | 103.6 | 109.2 | 1.66 |
| Pyr | 98.1 | 107.9 | 103.9 | 1.47 |
| BaA | 95.8 | 100.8 | 104.9 | 1.03 |
| Chr | 90.8 | 98.7 | 103.0 | 1.23 |
| BbF | 85.8 | 92.2 | 104.1 | 2.02 |
| BkF | 102.9 | 94.5 | 105.5 | 1.30 |
| Bap | 83.3 | 95.6 | 86.3 | 1.15 |

**Table S7.** Recovery rate and method detection limit (MDL) of NBs.

| **Compound** | **Recovery rate (%)** | | | **MDL (ng/L)** |
| --- | --- | --- | --- | --- |
|  | **20 ng/L** | **100 ng/L** | **200 ng/L** |  |
| NB | 84.0 | 85.6 | 85.0 | 2.29 |
| 2-NT | 83.1 | 87.2 | 78.6 | 5.91 |
| 3-NT | 83.1 | 77.4 | 74.0 | 2.66 |
| 4-NT | 81.3 | 86.8 | 80.4 | 5.30 |
| 1-C-3-NB | 82.1 | 83.8 | 70.1 | 4.12 |
| 1-C-2-NB | 85.3 | 81.2 | 71.2 | 4.96 |
| 1,4-DNB | 94.7 | 96.7 | 93.9 | 6.98 |
| 1,3-DNB | 94.6 | 95.2 | 105.6 | 9.28 |
| 2,6-DNT | 65.8 | 93.5 | 105.3 | 10.73 |
| 1,2-DNB | 100.4 | 93.2 | 96.1 | 22.74 |
| 2,4-DNT | 76.9 | 103.4 | 114.1 | 10.26 |
| 1-C-2,4-DNB | 71.3 | 81.8 | 80.9 | 13.16 |
| 3,4-DNT | 89.5 | 94.2 | 101.4 | 10.22 |
| TNT | 80.7 | 90.7 | 86.2 | 15.08 |

**Table S8.** Acute and chronic toxicity data and PNEC for 43 compounds.

| **Compound** | **Acute toxicity - E(L)C50 (mg/l)** | | | **Chronic toxicity - NOEC (mg/l)** | | | **AF** | **PNEC  (ng/l)** |
| --- | --- | --- | --- | --- | --- | --- | --- | --- |
|  | **Fish** | **fleas** | **large algae** | **Fish** | **fleas** | **large algae** |  |  |
| PCB28 | **0.16** | **0.16** | / | **0.016** | / | / | 100 | **160.00** |
| PCB52 | **0.03** | **0.03** | / | **0.025** | / | / | 100 | **250.00** |
| PCB101 | / | **0.01** | / | **0.025** | / | / | 100 | **250.00** |
| PCB81 | **0.015585** | / | / | / | / | / | 1000 | **15.59** |
| PCB77 | **0.002** | **0.002** | / | **0.1** | / | / | 100 | **1000.00** |
| PCB123 | 0.0217 | 0.0053 | / | / | / | / | 1000 | 5.30 |
| PCB118 | 0.0218 | 0.0054 | / | / | / | / | 1000 | 5.40 |
| PCB114 | 0.0218 | 0.00654 | / | / | / | / | 1000 | 6.54 |
| PCB138 | / | / | / | **0.025** | / | / | 100 | **250.00** |
| PCB105 | / | / | **0.00457** | **/** | / | / | 1000 | **4.57** |
| PCB153 | **0.0013** | **0.0013** | / | **0.025** | / | / | 100 | **250.00** |
| PCB126 | **0.00017** | / | / | **0.0005** | / | / | 100 | **5.00** |
| PCB167 | 0.00615 | 0.00308 | / | / | / | / | 1000 | 3.08 |
| PCB156 | 0.00585 | 0.00281 | / | / | / | / | 1000 | 2.81 |
| PCB157 | 0.00587 | 0.00281 | / | / | / | / | 1000 | 2.81 |
| PCB180 | / | / | / | **0.025** | / | / | 100 | **250.00** |
| Nap | **0.11** | **1.6** | **2.82** | **0.115356** | **0.6** | **4.15** | 10 | **11535.62** |
| Acy | 0.23 | 3.68 | / | / | / | / | 1000 | 230.00 |
| Ace | **0.58** | **0.12** | **0.322** | **0.033** | **0.026** | **0.1** | 10 | **2600.00** |
| Flu | **0.76** | **0.212** | **3.4** | **0.125** | **0.0625** | **1.67** | 10 | **6250.00** |
| Phe | **0.03** | **0.1** | **0.18** | **0.005** | **0.022279** | **2.74** | 10 | **500.00** |
| Ant | **0.00127** | **0.02** | **0.0033** | **0.00608** | **0.022279** | **0.003** | 10 | **300.00** |
| Flua | **0.0001** | **0.004** | **0.00538** | **0.0001** | **0.0014** | **0.0417** | 10 | **10.00** |
| Pyr | **0.2** | **0.004** | **0.007038** | **0.0152** | / | **1.44** | 50 | **304.00** |
| BaA | / | **0.000959** | **0.002625** | / | / | / | 1000 | **0.96** |
| Chr | / | **1.9** | / | / | / | / | 1000 | **1900.00** |
| BbF | / | / | / | / | / | **0.00159** | 100 | **15.90** |
| BkF | / | / | **0.003331** | **0.01** | / | / | 100 | **100.00** |
| Bap | **0.007112** | **0.000982** | **0.000631** | **0.00017** | **2.00E-05** | / | 50 | **0.40** |
| NB | **1.907** | **7.2** | **3.2** | **5** | **0.46** | **1.2** | 10 | **46000.00** |
| 2-NT | **30.1** | **1.012** | **22** | / | / | **4.4** | 100 | **44000.00** |
| 3-NT | **0.352** | **0.845** | **14** | **1.26** | / | / | 100 | **12600.00** |
| 4-NT | **0.684** | **0.407** | **22** | **/** | / | / | 1000 | **407.00** |
| 1-C-3-NB | **1.2** | **20** | **1.9** | **/** | / | / | 1000 | **1200.00** |
| 1-C-2-NB | **15** | **2.7** | **4.9** | **1.53** | **0.19** | / | 50 | **3800.00** |
| 1,4-DNB | 4.55 | 7.04 | / | / | / | / | 1000 | 4550.00 |
| 1,3-DNB | 8.92 | 3.93 | / | / | / | / | 1000 | 3930.00 |
| 2,6-DNT | **0.234** | **0.067** | **2.19** | **13.2** | **0.06** | / | 50 | **1200.00** |
| 1,2-DNB | **0.019** | **0.037** | / | / | / | / | 1000 | **19.00** |
| 2,4-DNT | **0.113** | **14** | **0.08** | **0.77** | **0.02** | / | 50 | **400.00** |
| 1-C-2,4-DNB | / | **0.8** | **0.8** | **/** | / | / | 1000 | **800.00** |
| 3,4-DNT | **1.5** | **3.1** | / | / | / | / | 1000 | **1500.00** |
| TNT | **0.8** | **0.98** | **0.567833** | **0.005** | **0.48** | **0.82** | 10 | **500.00** |

Note: The above data with rough marks are experimental data, and the data without rough marks are obtained from simulation calculation.

**Table S9.** 41 alternative indicators of plankton and their interference response

| **Attribute** | **Number** | **Parameter** | **Response to degradation** |
| --- | --- | --- | --- |
| Species richness | M1 | Phytoplankton species | Decrease |
|  | M2 | Bacillariophyta species | Decrease |
|  | M3 | Cyanophyta species | Decrease |
|  | M4 | Chlorophyta species | Decrease |
|  | M5 | Phytoplankton abundance | Decrease |
|  | M6 | Bacillariophyta abundance | Decrease |
|  | M7 | Cyanophyta abundance | Increase |
|  | M8 | Chlorophyta abundance | Decrease |
|  | M9 | Bloom algal abundance | Decrease |
|  | M10 | Abundance of the top three dominant species of phytoplankton | Increase |
|  | M11 | Zooplankton species | Decrease |
|  | M12 | Rotifera species | Decrease |
|  | M13 | Cladocera species | Decrease |
|  | M14 | Copepoda species | Decrease |
|  | M15 | Zooplankton abundance | Decrease |
|  | M16 | Rotifera abundance | Increase |
|  | M17 | Cladocera abundance | Decrease |
|  | M18 | Copepoda abundance | Decrease |
|  | M19 | Abundance of the top three dominant species of zooplankton | Increase |
| Community structure | M20 | Percentage of Bacillariophyta species | Increase |
| composition | M21 | Percentage of Cyanophyta species | Increase |
|  | M22 | Percentage of Chlorophyta species | Decrease |
|  | M23 | Relative abundance of Bacillariophyta | Decrease |
|  | M24 | Relative abundance of Cyanophyta | Increase |
|  | M25 | Relative abundance of Chlorophyta | Decrease |
|  | M26 | Relative abundance of bloom algal | Decrease |
|  | M27 | Relative abundance of the top three dominant species of phytoplankton | Increase |
|  | M28 | Centricae abundance / Pennatae abundance ratio | Increase |
|  | M29 | Percentage of Rotifera species | Increase |
|  | M30 | Percentage of Cladocera species | Decrease |
|  | M31 | Percentage of Copepoda species | Decrease |
|  | M32 | Relative abundance of Rotifera | Increase |
|  | M33 | Relative abundance of Cladocera | Decrease |
|  | M34 | Relative abundance of Copepoda | Decrease |
|  | M35 | Relative abundance of the top three dominant species of zooplankton | Increase |
| Community diversity | M36 | The Shannon-Wiener index (*H’*) of Phytoplankton | Decrease |
|  | M37 | The Pielou index (*J*) of Phytoplankton | Decrease |
|  | M38 | The Margalef index (*d*)of Phytoplankton | Decrease |
|  | M39 | The Shannon-Wiener index (*H’*) of zooplankton | Decrease |
|  | M40 | The Pielou index (*J*) of zooplankton | Decrease |
|  | M41 | The Margalef index (*d*) of zooplankton | Decrease |

Note: Bloom algae refers to *Microcystis sp.*, *Anabeana sp.*, and *Sargassum sp.*

**Table S10.** Correlation analysis results of 23 alternative indicators in the lower reaches of the Yangtze River

| **指标** | **M1** | **M2** | **M4** | **M6** | **M8** | **M11** | **M12** | **M14** | **M18** | **M20** | **M22** | **M25** | **M27** | **M28** | **M32** | **M34** | **M35** | **M36** | **M37** | **M38** | **M39** | **M40** | **M41** |
| --- | --- | --- | --- | --- | --- | --- | --- | --- | --- | --- | --- | --- | --- | --- | --- | --- | --- | --- | --- | --- | --- | --- | --- |
| **M1** | 1 |  |  |  |  |  |  |  |  |  |  |  |  |  |  |  |  |  |  |  |  |  |  |
| **M2** | **0.872^**^** | 1 |  |  |  |  |  |  |  |  |  |  |  |  |  |  |  |  |  |  |  |  |  |
| **M4** | **0.902^**^** | 0.619^**^ | 1 |  |  |  |  |  |  |  |  |  |  |  |  |  |  |  |  |  |  |  |  |
| **M6** | 0.332^**^ | 0.100 | 0.394^**^ | 1 |  |  |  |  |  |  |  |  |  |  |  |  |  |  |  |  |  |  |  |
| **M8** | 0.227^*^ | 0.119 | 0.244^*^ | **0.775^**^** | 1 |  |  |  |  |  |  |  |  |  |  |  |  |  |  |  |  |  |  |
| **M11** | **0.792^**^** | 0.674^**^ | 0.723^**^ | 0.221^*^ | 0.152 | 1 |  |  |  |  |  |  |  |  |  |  |  |  |  |  |  |  |  |
| **M12** | **0.797^**^** | **0.718^**^** | 0.713^**^ | 0.159 | 0.088 | **0.946^**^** | 1 |  |  |  |  |  |  |  |  |  |  |  |  |  |  |  |  |
| **M14** | 0.487^**^ | 0.287^**^ | 0.496^**^ | 0.288^**^ | 0.190 | 0.748^**^ | 0.540^**^ | 1 |  |  |  |  |  |  |  |  |  |  |  |  |  |  |  |
| **M18** | 0.538^**^ | 0.504^**^ | 0.449^**^ | 0.029 | 0.017 | 0.678^**^ | 0.613^**^ | 0.632^**^ | 1 |  |  |  |  |  |  |  |  |  |  |  |  |  |  |
| **M20** | -0.364^**^ | 0.098 | -0.587^**^ | -0.438^**^ | -0.192 | -0.301^**^ | -0.223^*^ | -0.424^**^ | -0.139 | 1 |  |  |  |  |  |  |  |  |  |  |  |  |  |
| **M22** | 0.606^**^ | 0.249^*^ | **0.851^**^** | 0.376^**^ | 0.209 | 0.503^**^ | 0.458^**^ | 0.433^**^ | 0.291^**^ | -0.746^**^ | 1 |  |  |  |  |  |  |  |  |  |  |  |  |
| **M25** | 0.078 | 0.079 | 0.141 | -0.043 | 0.285^**^ | 0.283^**^ | 0.284^**^ | 0.139 | 0.035 | 0.063 | 0.144 | 1 |  |  |  |  |  |  |  |  |  |  |  |
| **M27** | -0.513^**^ | -0.483^**^ | -0.474^**^ | 0.093 | 0.043 | -0.389^**^ | -0.366^**^ | -0.262^*^ | -0.421^**^ | 0.049 | -0.325^**^ | -0.106 | 1 |  |  |  |  |  |  |  |  |  |  |
| **M28** | -0.459^**^ | -0.535^**^ | -0.314^**^ | 0.147 | 0.046 | -0.356^**^ | -0.360^**^ | -0.144 | -0.386^**^ | -0.106 | -0.063 | -0.120 | 0.526^**^ | 1 |  |  |  |  |  |  |  |  |  |
| **M32** | -0.615^**^ | -0.674^**^ | -.504^**^ | 0.000 | -0.113 | -0.605^**^ | -0.557^**^ | -0.404^**^ | -0.641^**^ | -0.084 | -0.293^**^ | -0.148 | 0.624^**^ | 0.483^**^ | 1 |  |  |  |  |  |  |  |  |
| **M34** | 0.575^**^ | 0.506^**^ | 0.562^**^ | 0.155 | 0.149 | 0.639^**^ | 0.576^**^ | 0.534^**^ | 0.686^**^ | -0.077 | 0.377^**^ | 0.216^*^ | -0.576^**^ | -0.382^**^ | **-0.854^**^** | 1 |  |  |  |  |  |  |  |
| **M35** | -0.424^**^ | -0.482^**^ | -0.354^**^ | -0.072 | -0.123 | -0.424^**^ | -0.388^**^ | -0.335^**^ | -0.413^**^ | -0.109 | -0.197 | -0.130 | 0.491^**^ | 0.383^**^ | 0.731^**^ | -0.659^**^ | 1 |  |  |  |  |  |  |
| **M36** | **0.889^**^** | **0.808^**^** | **0.784^**^** | 0.127 | 0.076 | 0.730^**^ | 0.710^**^ | 0.477^**^ | 0.585^**^ | -0.276^**^ | 0.516^**^ | 0.023 | -0.673^**^ | -0.435^**^ | -0.652^**^ | 0.564^**^ | -0.430^**^ | 1 |  |  |  |  |  |
| **M37** | 0.430^**^ | 0.396^**^ | 0.404^**^ | -0.159 | -0.144 | 0.385^**^ | 0.340^**^ | 0.300^**^ | 0.408^**^ | -0.097 | 0.259^*^ | -0.036 | -0.703^**^ | -0.259^*^ | -0.490^**^ | 0.432^**^ | -0.325^**^ | **0.775^**^** | 1 |  |  |  |  |
| **M38** | **0.996^**^** | **0.890^**^** | **0.888^**^** | 0.257^*^ | 0.169 | **0.800^**^** | **0.807^**^** | 0.480^**^ | 0.557^**^ | -0.321^**^ | 0.584^**^ | 0.092 | -0.539^**^ | -0.477^**^ | -0.646^**^ | 0.593^**^ | -0.434^**^ | **0.910^**^** | 0.474^**^ | 1 |  |  |  |
| **M39** | 0.610^**^ | 0.566^**^ | 0.516^**^ | 0.243^*^ | 0.239^*^ | **0.818^**^** | **0.761^**^** | 0.582^**^ | 0.515^**^ | -0.128 | 0.328^**^ | 0.306^**^ | -0.251^*^ | -0.263^*^ | -0.569^**^ | 0.580^**^ | -0.506^**^ | 0.567^**^ | 0.292^**^ | 0.618^**^ | 1 |  |  |
| **M40** | 0.100 | 0.143 | 0.051 | 0.161 | 0.201 | 0.202 | 0.167 | 0.143 | 0.082 | 0.150 | -0.006 | 0.122 | -0.070 | -0.078 | -0.286^**^ | 0.335^**^ | -0.455^**^ | 0.089 | 0.069 | 0.102 | 0.683^**^ | 1 |  |
| **M41** | 0.543^**^ | 0.468^**^ | 0.527^**^ | 0.139 | 0.161 | **0.783^**^** | 0.738^**^ | 0.528^**^ | 0.351^**^ | -0.139 | 0.367^**^ | 0.533^**^ | -0.201 | -0.206 | -0.505^**^ | 0.567^**^ | -0.359^**^ | 0.450^**^ | 0.214^*^ | 0.558^**^ | **0.766^**^** | 0.368^**^ | 1 |

Note: "*" indicates *p* < 0.05, "**" indicates *p* < 0.01.

**Table S11.** Calculation standard of ratio method for core indicators

| **Number** | **Core index** | **Response to degradation** | **Calculation standard** |
| --- | --- | --- | --- |
| M6 | Bacillariophyta abundance | Decrease | M6/979370 |
| M14 | Copepoda species | Decrease | M14/6.5 |
| M18 | Copepoda abundance | Decrease | M18/151.48 |
| M20 | Percentage of Bacillariophyta species | Increase | (88.89-M20) / (88.89-39.23) |
| M22 | Percentage of Chlorophyta species | Decrease | M22/38.35 |
| M25 | Relative abundance of Chlorophyta | Decrease | M25/58.41 |
| M27 | Relative abundance of the top three dominant species of phytoplankton | Increase | (92.20-M27) / (92.20-25.6) |
| M28 | Centricae abundance / Pennatae abundance ratio | Increase | (54.50-M28) / (54.50-1.64) |
| M32 | Relative abundance of Rotifera | Increase | (99.69-M32) / (99.69-7.48) |
| M35 | Relative abundance of the top three dominant species of zooplankton | Increase | (99.01-M35) / (99.01-9.92) |
| M36 | The Shannon-Wiener index (*H’*) of Phytoplankton | Decrease | M36/2.82 |
| M39 | The Shannon-Wiener index (*H’*) of zooplankton | Decrease | M39/2.28 |
| M40 | The Pielou index (*J*) of zooplankton | Decrease | M40/0.85 |

**Table S12.** Summary of Reported PCBs, PAHs, and NBs concentrations in surface water around the world.

|  | **N** | **Location** | **year (s)** | **Range (ng/L)** | **Mean** | **Reference** |
| --- | --- | --- | --- | --- | --- | --- |
| **PCBs** | 5 | Yangtze River (Jiangsu), China | 2004–2005 | <0.21–25.7 | / | He and Hu et al., 2011 |
|  | 43 | Songhua River, China | 2007–2008 | 1.1–14 | / | (You and Ding et al., 2011) |
|  | 38 | the Yangtze River Delta, China | 2009 | 1.23–16.6 | / | (Zhang and Shi et al., 2011) |
|  | 7 | Yangtze River, China | 2016 | 0.005–0.38 | / | (Cui and Dong et al., 2020) |
|  | 9 | Moscow River, Russia | 2013 | ND–180.7 | / | (Eremina and Paschke et al., 2016) |
|  | 32 | Volturno River, Italy | 2017–2018 | 2.28–10 | 6.07 | (Montuori and De Rosa et al., 2020) |
|  | 7 | the Atlantic Iberian northwest coastline | 2018–2019 | ND–3 | / | (Rocha and Ribeiro et al., 2021) |
|  | 9 | Nairobi River, Kenya | 2008 | ND–718.78 | / | (Ndunda and Wandiga, 2020) |
| **PAHs** | 16 | Songhua River, China | 2007–2008 | 163.54–2746.25 | 934.62 | (Zhao and Ding et al., 2014) |
|  | 15 | Qiantang River, China | 2005 | 70.3–1844.0 | 283.3 | (Chen and Zhu et al., 2007) |
|  | 16 | Yellow River, China | 2005–2006 | 144.3–2,361 | 662 | (Sun and Wang et al., 2009) |
|  | 16 | Jiulong River, China | 2010–2011 | 19.1–125.9 | / | (Wu and Wang et al., 2019) |
|  | 16 | Huaihe River, China | 2013–2014 | 79.94–421.07 | 140.37 | (Liu and Feng et al., 2016) |
|  | 15 | the Yangtze River Estuary, China | 2014-2015 | 38.6–90.5 | 61.4 | (Jiang and Lin et al., 2018) |
|  | 16 | the Three Gorges Reservoir, China | 2015 | ND–42.9 | / | (Zheng and Ma et al., 2016) |
|  | 16 | the middle-lower Yangtze River, China | 2017–2018 | 2.4–761.2 | 73.7 | (Zhao and Gong et al., 2021) |
|  | 16 | the River Nile system, Egypt | 2005 | 1112.7–4351.2 | / | (Badawy and Embaby, 2010) |
|  | 14 | the Almendares River, Cuba | 2005 | 836.0–15,811.0 | 2512 | (Santana and Massone et al., 2015) |
|  | 16 | the Elbe and Weser River, Germany | 2010 | 10.0–40.0 | / | (Siemers and Mänz et al., 2015) |
|  | 16 | a Typical Upland Stream, U.K. | 2010–2011 | 2.7–20.0 | / | (Moeckel and Monteith et al., 2014) |
|  | 13 | Moscow River, Russia | 2013 | 50.6–120.1 | / | (Eremina and Paschke et al., 2016) |
| **NBs** | 10 | the middle-lower Yangtze River, China | 2004 | 269–9052 | 1726 | (He and Sun et al., 2006) |
|  | 8 | Liao River, China | 2006 | 726–15560 | 3402 | (Men and Wang et al., 2011) |





**Fig. S1.** The boxplot for the discriminant ability test of 31 alternative indicators in the lower reaches of the Yangtze River.


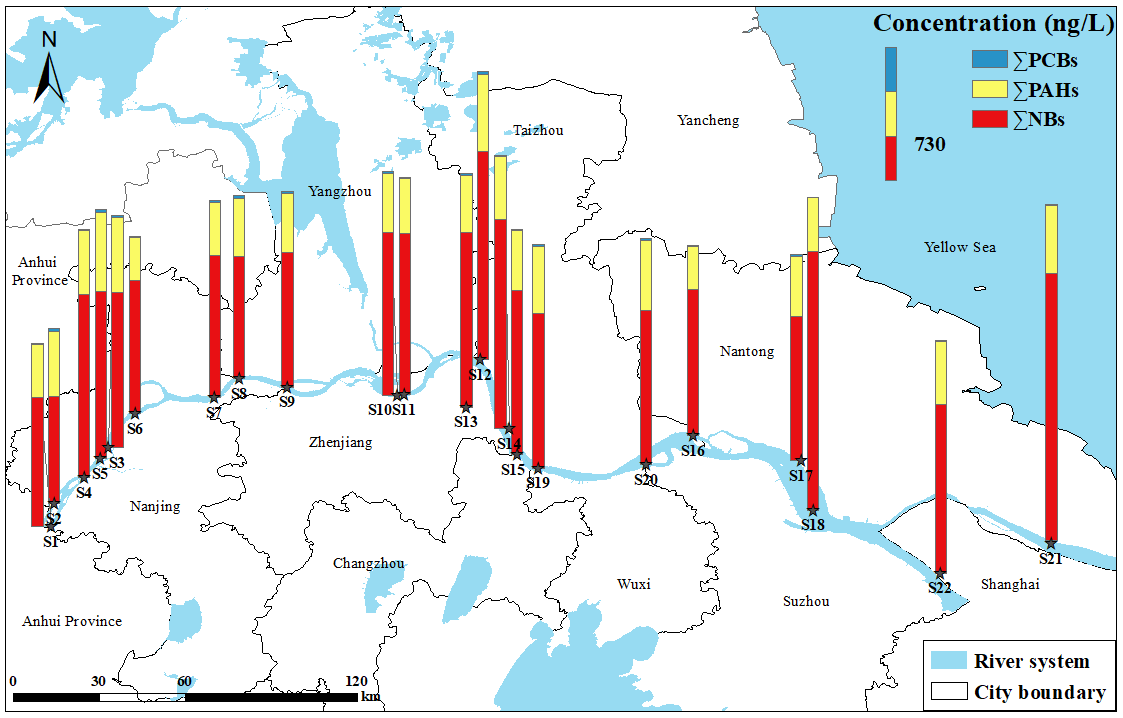


**Fig. S2.** Concentration distribution of PCBs, PAHs, and NBs in the lower reaches of the Yangtze River.


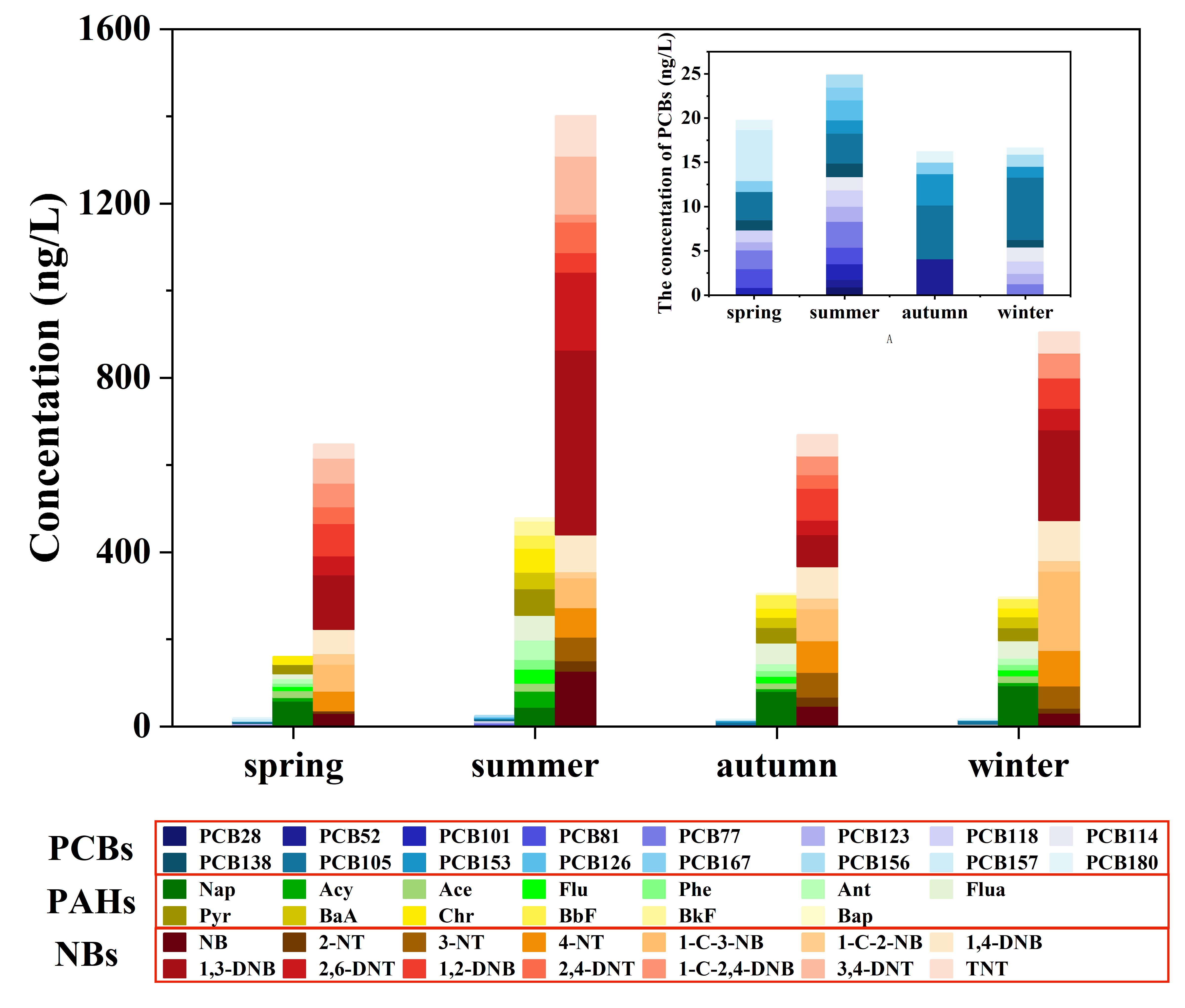


**Fig. S3.** Cumulative bar chart of the concentrations of 43 compounds in all season. The data for each season are divided into three categories, including PCBs, PAHs and NBs.


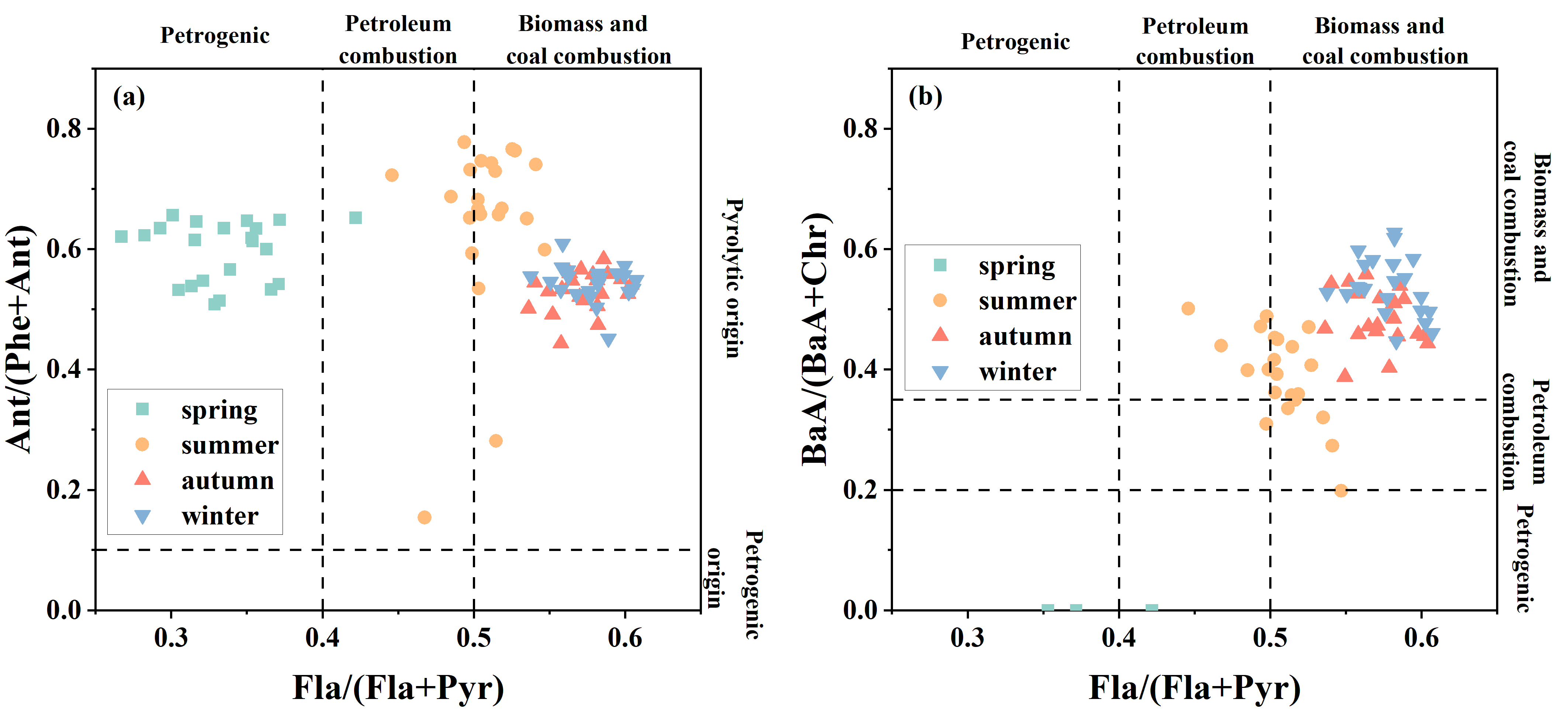


**Fig. S4.** Cross plots for the isomeric ratios of Fla/ (Fla + Pyr) versus Ant / (Ant + Phe) (a), and Fla/ (Fla + Pyr) versus BaA / (BaA + Chr) (b).


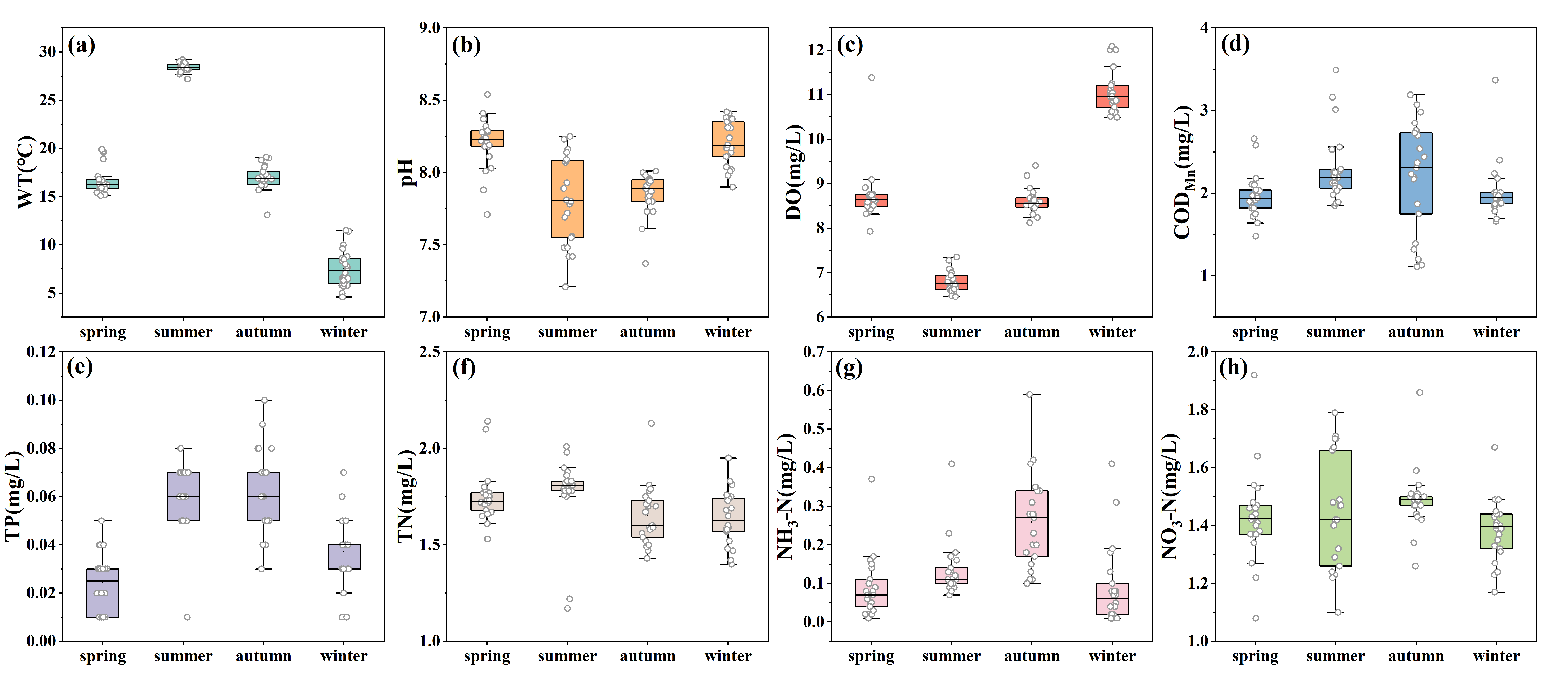


**Fig. S5.** Seasonal distribution of conventional index (a: WT, b: pH, c: DO, d: COD_Mn_, e: TP, f: TN, g: NH_3_-N, h: NO_3_-N) in the LYR.


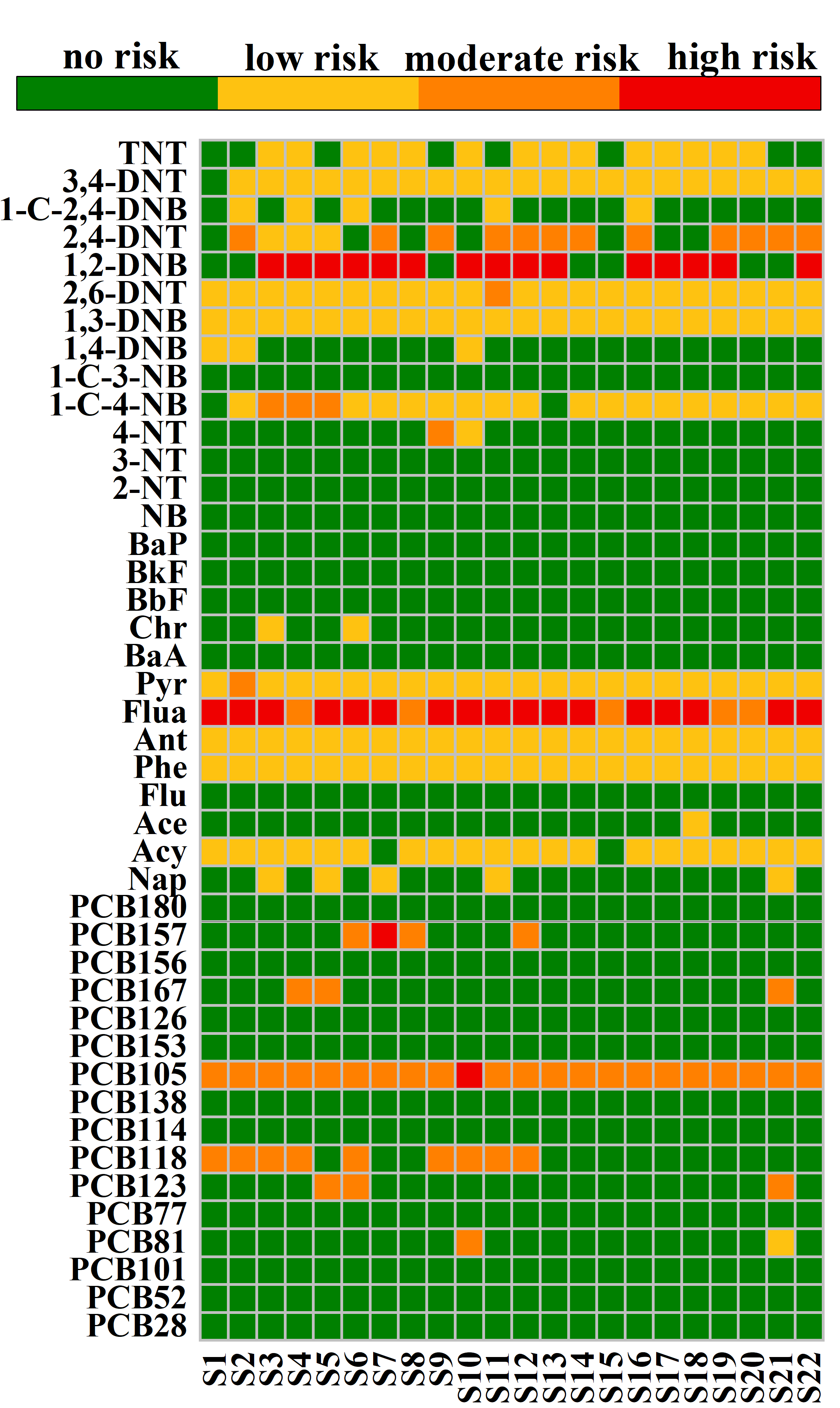


**Fig. S6.** RQ value of PCBs, PAHs and NBs in Spring in the lower reaches of the Yangtze River.


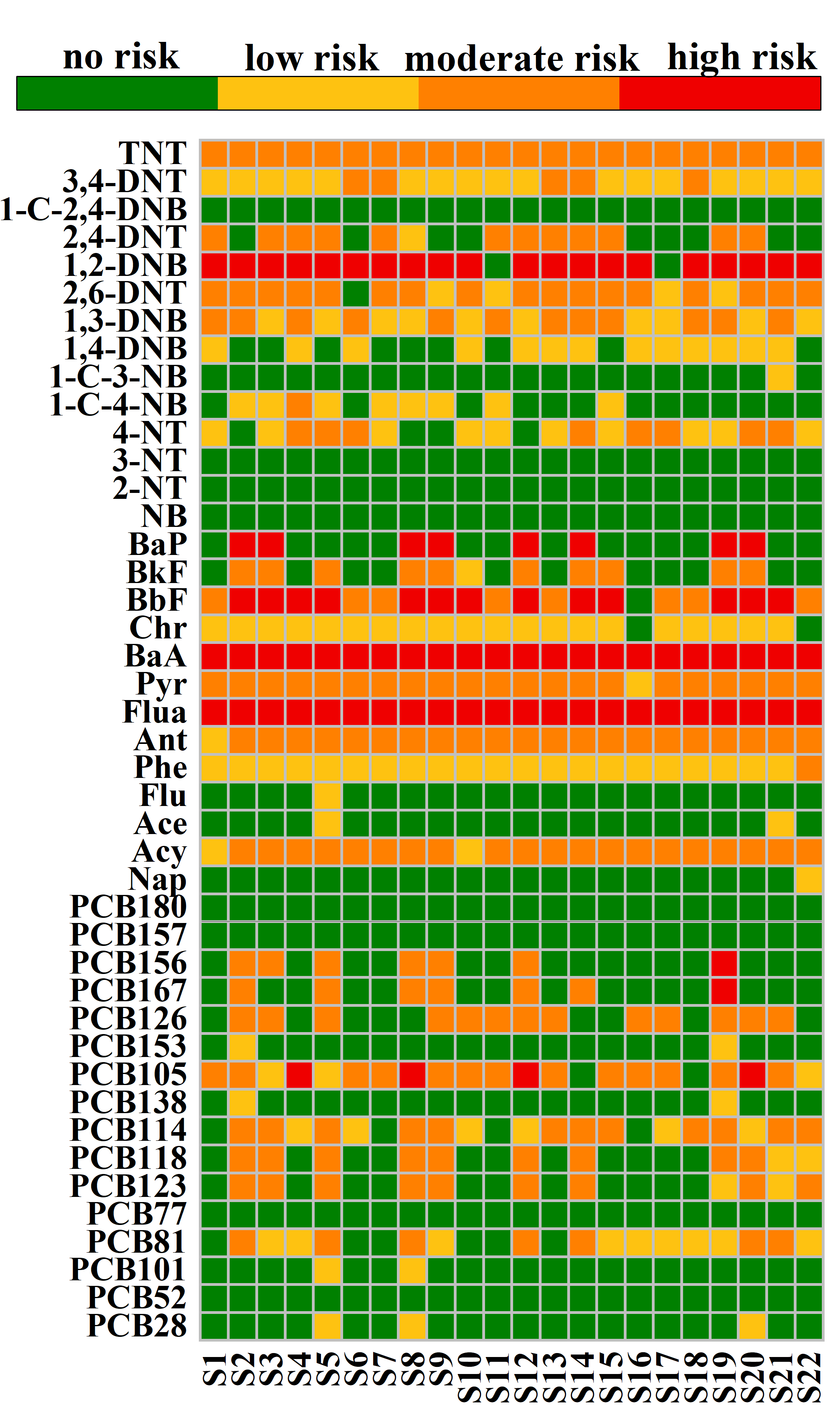


**Fig. S7.** RQ value of PCBs, PAHs and NBs in Summer in the lower reaches of the Yangtze River.


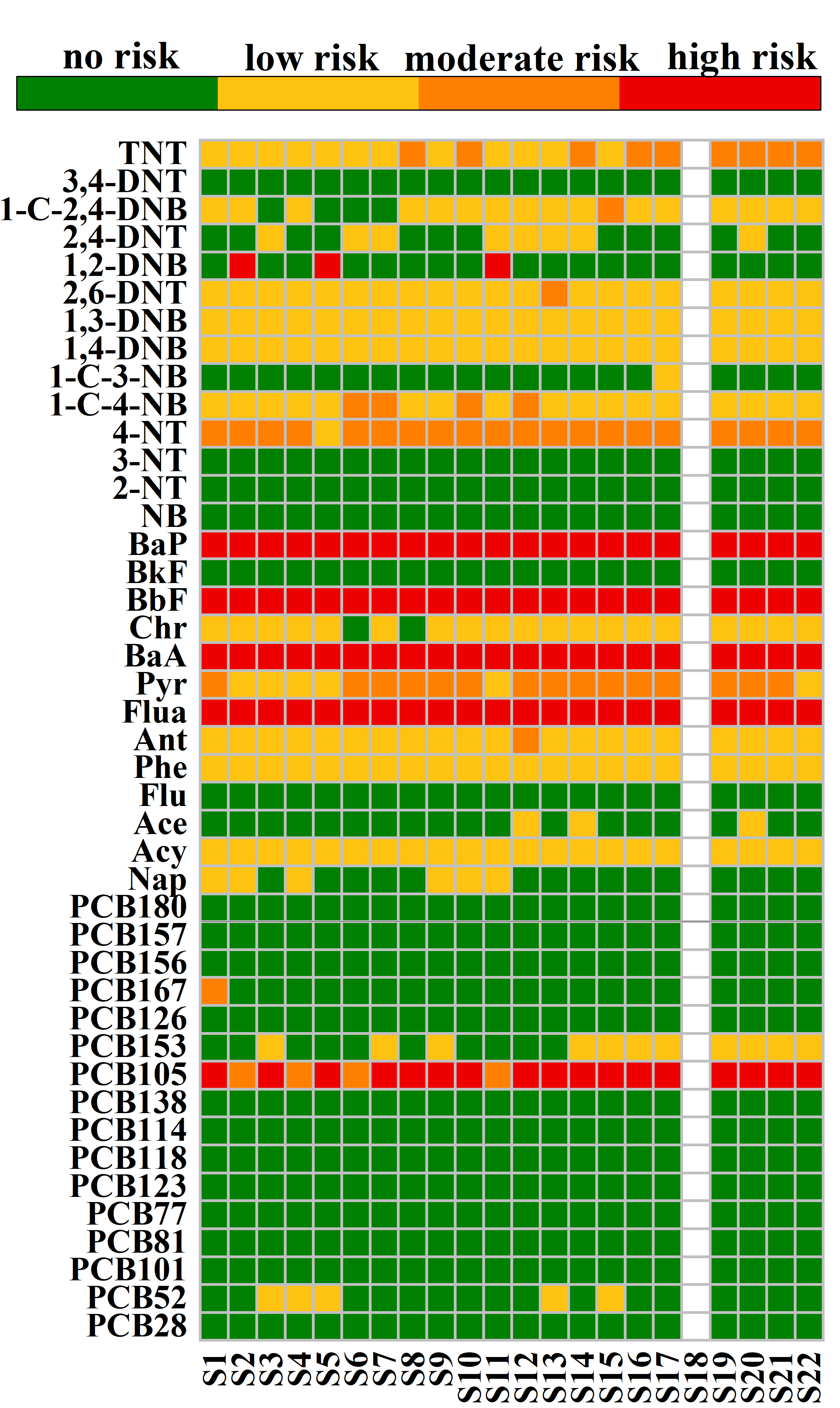


**Fig. S8.** RQ value of PCBs, PAHs and NBs in Autumn in the lower reaches of the Yangtze River.


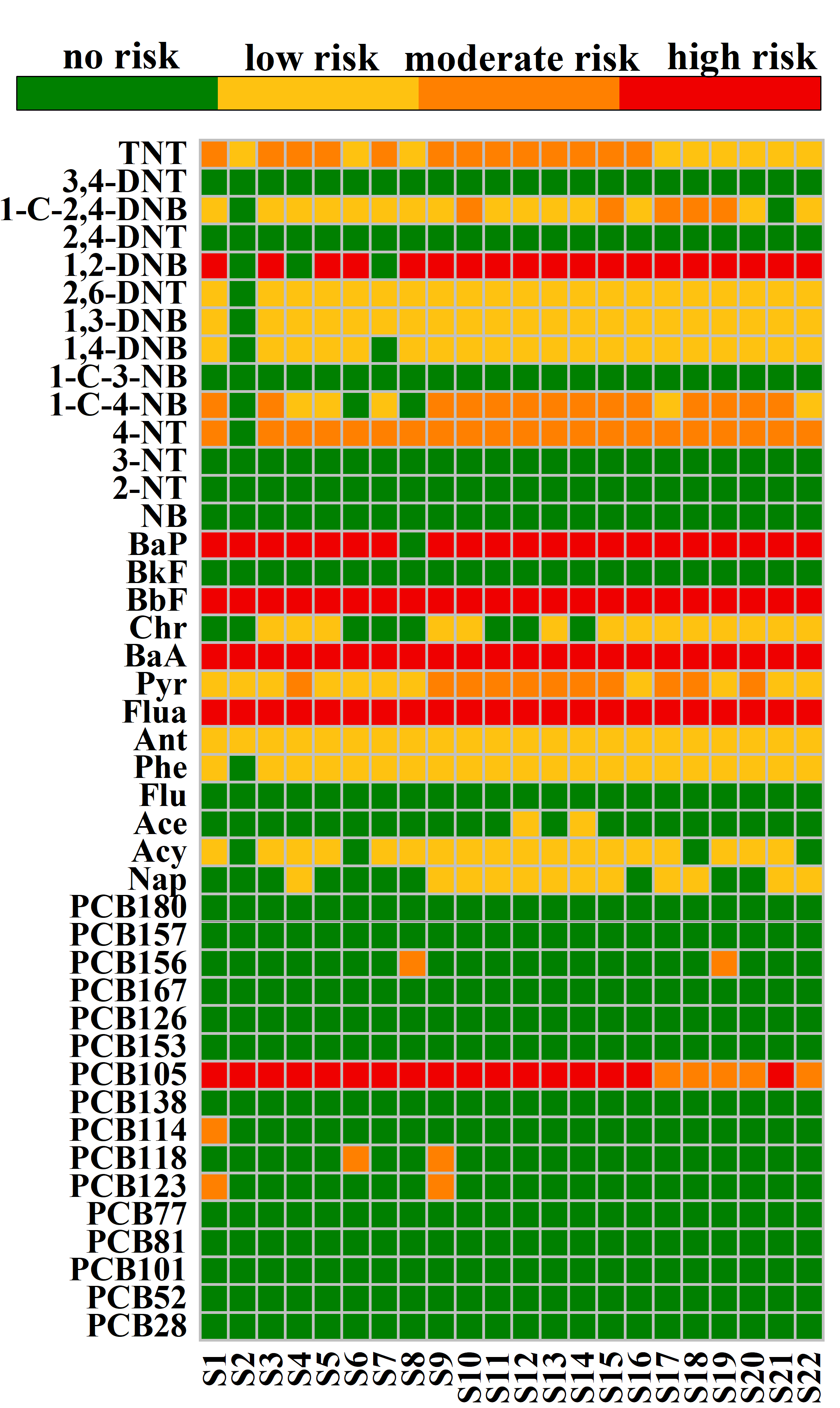


**Fig. S9.** RQ value of PCBs, PAHs and NBs in Winter in the lower reaches of the Yangtze River.


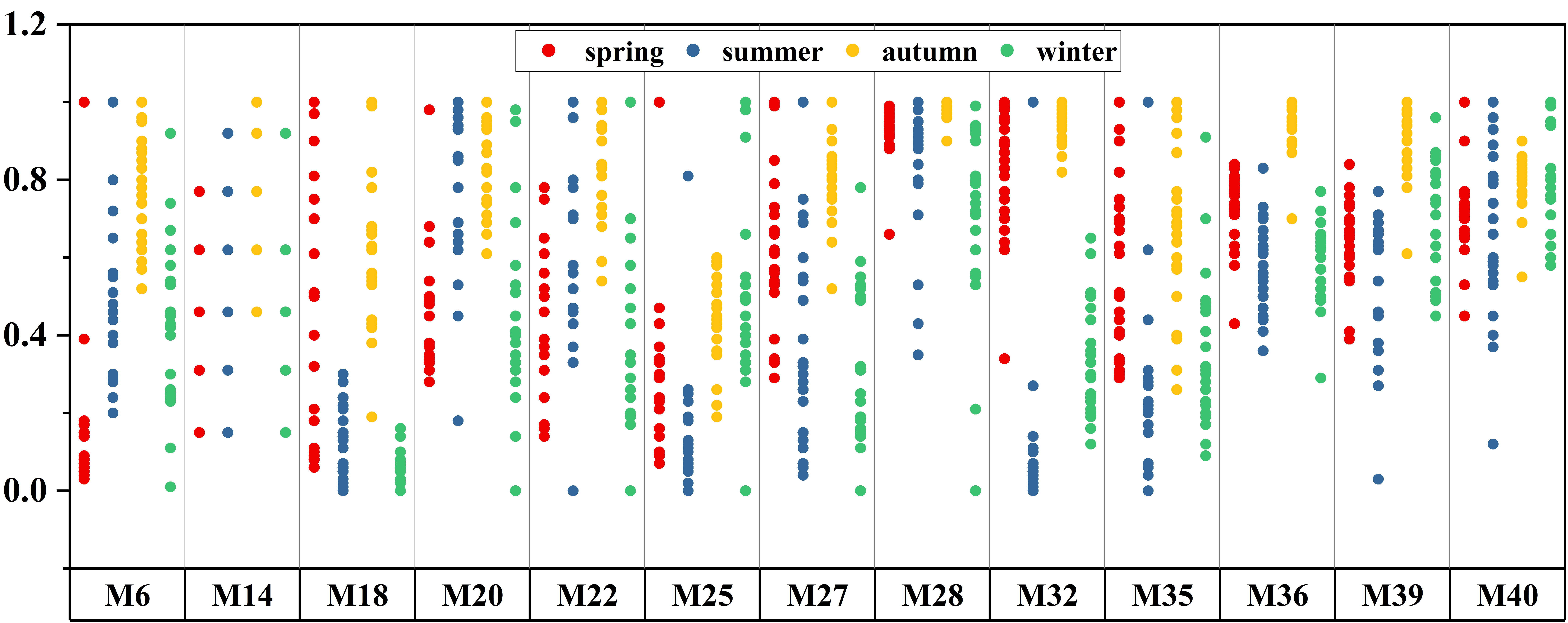


**Fig. S10.** Seasonal distribution of 13 core Index scores

**References:**

Badawy, M. I. and M. A. Embaby (2010). Distribution of polycyclic aromatic hydrocarbons in drinking water in Egypt. Desalination **251** (1–3): 34–40.

Chen, Y. and L. Zhu, et al. (2007). Characterization and distribution of polycyclic aromatic hydrocarbon in surface water and sediment from Qiantang River, China. Journal of Hazardous Materials **141** (1): 148–155.

Cui, X. and J. Dong, et al. (2020). Polychlorinated biphenyls in the drinking water source of the Yangtze River: characteristics and risk assessment. Environmental Sciences Europe **32** (1).

Eremina, N. and A. Paschke, et al. (2016). Distribution of polychlorinated biphenyls, phthalic acid esters, polycyclic aromatic hydrocarbons and organochlorine substances in the Moscow River, Russia. Environmental Pollution **210**: 409–418.

He, H. and G. Hu, et al. (2011). Trace analysis of persistent toxic substances in the main stream of Jiangsu section of the Yangtze River, China. Environmental Science and Pollution Research **18** (4): 638–648.

He, M. and Y. Sun, et al. (2006). Distribution patterns of nitrobenzenes and polychlorinated biphenyls in water, suspended particulate matter and sediment from mid- and down-stream of the Yellow River (China). Chemosphere **65** (3): 365–374.

Jiang, Y. and T. Lin, et al. (2018). Seasonal atmospheric deposition and air–sea gas exchange of polycyclic aromatic hydrocarbons over the Yangtze River Estuary, East China Sea: Implications for source–sink processes. Atmospheric Environment **178**: 31–40.

Liu, M. and J. Feng, et al. (2016). Spatial-temporal distributions, sources of polycyclic aromatic hydrocarbons (PAHs) in surface water and suspended particular matter from the upper reach of Huaihe River, China. Ecological Engineering **95**: 143–151.

Men, B. and H. Wang, et al. (2011). Distribution patterns of nitroaromatic compounds in the water, suspended particle and sediment of the river in a long-term industrial zone (China)." Environmental Monitoring and Assessment **177** (1-4): 515–526.

Moeckel, C. and D. T. Monteith, et al. (2014). Relationship between the Concentrations of Dissolved Organic Matter and Polycyclic Aromatic Hydrocarbons in a Typical U.K. Upland Stream. Environmental Science & Technology **48** (1): 130–138.

Montuori, P. and E. De Rosa, et al. (2020). Polychlorinated biphenyls and organochlorine pesticides in water and sediment from Volturno River, Southern Italy: occurrence, distribution and risk assessment. Environmental Sciences Europe **32** (1).

Ndunda, E. N. and S. O. Wandiga (2020). Spatial and temporal trends of polychlorinated biphenyls in water and sediment from Nairobi River, Kenya. Environmental Monitoring and Assessment **192** (9).

Rocha, M. J. and A. B. Ribeiro, et al. (2021). Temporal-spatial survey of PAHs and PCBs in the Atlantic Iberian northwest coastline, and evaluation of their sources and risks for both humans and aquatic organisms. Chemosphere **279**: 130506.

Santana, J. L. and C. G. Massone, et al. (2015). Occurrence and Source Appraisal of Polycyclic Aromatic Hydrocarbons (PAHs) in Surface Waters of the Almendares River, Cuba. Archives of Environmental Contamination and Toxicology **69** (2): 143–152.

Siemers, A. and J. S. Mänz, et al. (2015). Development and application of a simultaneous SPE-method for polycyclic aromatic hydrocarbons (PAHs), alkylated PAHs, heterocyclic PAHs (NSO-HET) and phenols in aqueous samples from German Rivers and the North Sea. Chemosphere **122**: 105–114.

Sun, J. and G. Wang, et al. (2009). Distribution of polycyclic aromatic hydrocarbons (PAHs) in Henan Reach of the Yellow River, Middle China. Ecotoxicology and Environmental Safety **72** (5): 1614–1624.

Wu, Y. and X. Wang, et al. (2019). Seasonal variation and spatial transport of polycyclic aromatic hydrocarbons in water of the subtropical Jiulong River watershed and estuary, Southeast China. Chemosphere **234**: 215–223.

You, H. and J. Ding, et al. (2011). Spatial and seasonal variation of polychlorinated biphenyls in Songhua River, China. Environmental Geochemistry and Health **33** (3): 291–299.

Zhang, L. and S. Shi, et al. (2011). Concentrations and possible sources of polychlorinated biphenyls in the surface water of the Yangtze River Delta, China. Chemosphere **85** (3): 399–405.

Zhao, X. and J. Ding, et al. (2014). Spatial distribution and temporal trends of polycyclic aromatic hydrocarbons (PAHs) in water and sediment from Songhua River, China. Environmental Geochemistry and Health **36** (1): 131–143.

Zhao, Z. and X. Gong, et al. (2021). Riverine transport and water-sediment exchange of polycyclic aromatic hydrocarbons (PAHs) along the middle-lower Yangtze River, China. Journal of Hazardous Materials **403**: 123973.

Zheng, B. and Y. Ma, et al. (2016). Distribution, sources, and risk assessment of polycyclic aromatic hydrocarbons (PAHs) in surface water in industrial affected areas of the Three Gorges Reservoir, China. Environmental Science and Pollution Research **23** (23): 23485–23495.
